# Lipoprotein receptors in ovary of eel, *Anguilla australis*: molecular characterisation of putative vitellogenin receptors

**DOI:** 10.1007/s10695-023-01169-6

**Published:** 2023-01-17

**Authors:** Lucila Babio, Erin L. Damsteegt, P. Mark Lokman

**Affiliations:** Department of Zoology, University of Otago, 340 Great King Street, P.O. Box 56, Dunedin, Otago 9054 New Zealand

**Keywords:** Low-density lipoprotein receptor family, Vitellogenin receptor, Lr8, Lrp13, Vitellogenin uptake, Egg yolk formation

## Abstract

Lipoprotein receptors, including low-density lipoprotein receptor (LDLr) relatives (Lrs) and LDLr-related proteins (Lrps), belong to the LDLr supergene family and participate in diverse physiological functions. In this study, novel sequences of *lr* and *lrp* genes expressed in the ovary of the short-finned eel, *Anguilla australis*, during early gonadal development are presented. The genes encoding the LDLr-like, Lrp1-like, Lrp1b-like, Lrp3, Lrp4-like, Lrp5-like, Lrp6, Lrp10, Lrp11, Lrp12-like, and Lr11-like proteins were found and identified by sequence and structure analysis, in addition to phylogenetic analysis. Genes encoding proteins previously implicated in follicle development and vitellogenin (Vtg) uptake in oviparous vertebrates were also identified, i.e. *lr8* (including *lr8* + and *lr8-* variants) and *lrp13*; their identification was reinforced by conserved synteny with orthologues in other teleost fish. Compared to other *lr*/*lrp* genes, the genes encoding Lr8 + , Lr8-, and Lrp13 were highly expressed in ovary during early development, decreasing as oocyte development advanced when induced by hypophysation. Furthermore, *lr8* + , *lr8-*, and *lrp13* were dominantly expressed in the ovary when compared with 17 other tissues. Finally, this study successfully detected the expression of both *lr8* variants, which showed different expression patterns to those reported in other oviparous vertebrates and provided the first characterisation of Lrp13 in *Anguilla* sp. We propose that *lr8* + , *lr8-*, and* lrp13* encode putative Vtg receptors in anguillid eels.

**Supplementary Information:**

The online version contains supplementary material available at 10.1007/s10695-023-01169-6.

## Introduction

The low-density lipoprotein receptor (LDLr) family is represented by single-pass transmembrane proteins that share some distinctive common features with the first member discovered, the LDLr (also known as Lr7): (*i*) ligand-binding domains or regions with a member-specific number of LDL class A (LDLa) repeats containing the negatively charged DxSDE conserved sequence, (*ii*) epidermal growth factor (EGF) precursor homology domains with EGF-like repeats and YWTD repeats, and (*iii*) a cytoplasmic domain usually containing motifs for internalisation (e.g. FxNPxY), or for signalling (Babin et al. 2007; Schneider 2008). The family includes LDLr relatives (Lrs) and LDLr-related proteins (Lrps), of which core members in vertebrates are the LDLr and Lr8 (also known as very low-density lipoprotein receptor (VLDLr) and/or vitellogenin receptor (Vtgr)). It also includes Lrp1, Lrp1b, Lrp2, Lrp4, Lrp8, and Lrp13 (characterised in teleost fish: Reading et al. 2014), while more distant members are the Lrp3, Lrp5, Lrp6, Lrp10, Lrp11, Lrp12, and Lr11 (sorting protein-related receptor containing LDLr class A repeats (SorLA)) (Príncipe et al. 2021). Additionally, some members contain special features such as the *O*-linked sugar domains associated with the LDLr, Lr8, and Lrp8, or the distinct domains associated with more distant members, e.g. the vacuolar protein sorting-10 domain (VPS10) associated with Lr11, the CUB (for complement C1s/C1r, sea urchin epidermal growth factor (Uegf) and bone morphogenic protein-1 (Bmp1)) domain associated with the Lrp3, Lrp10, and Lrp12, or the MANEC domain associated with Lrp11.

In oviparous vertebrates, members of the LDLr family play important roles in oocyte development and growth, facilitating vitellogenin (Vtg) uptake. While Lr8 binds Vtg in frog (*Xenopus laevis*: Okabayashi et al. 1996), chicken (*Gallus gallus*, which also binds VLDL: Stifani et al. 1990) and various teleost fish (rainbow trout, *Oncorhynchus mykiss*: Davail et al. 1998; blue tilapia, *Oreochromis aureus*: Li et al. 2003; cutthroat trout, *Oncorhynchus clarki*: Mizuta et al. 2013; and white perch, *Morone americana*: Reading et al. 2014), recently, the novel Lrp13 member was characterised and confirmed to bind Vtg in white perch (Reading et al. 2014) and cutthroat trout (Mushirobira et al. 2015). Additionally, the *lr8* gene presents two transcript variants, *lr8* + and *lr8-*, that only differ in the presence or absence, respectively, of a region encoding an *O*-linked sugar domain (in frog: Okabayashi et al. 1996; chicken: Bujo et al. 1995; Senegalese sole, *Solea senegalensis*: Agulleiro et al. 2007; Atlantic salmon, *Salmo salar*: Andersen et al. 2017; blue tilapia: Li et al. 2003; cutthroat trout: Mizuta et al. 2013; rainbow trout: Prat et al. 1998; and possibly, in white perch: Hiramatsu et al. 2004; Reading et al. 2011, 2014). However, the Lr8- isoform has historically been considered the oocyte-specific Vtgr, while the Lr8 + isoform has been suggested to be a somatic receptor (Bujo et al. 1995; Mizuta et al. 2013; Prat et al. 1998).

The short-finned eel (SFE, *Anguilla australis*) is one of the two main temperate species of anguillid eels found in New Zealand. Anguillid eels represent a group of basal teleost fish, the Elopomorpha, which correspond to one of the earliest evolved group within the teleost lineage (Chen et al. 2015; Takezaki 2021). As such, from an evolutionary perspective, its physiology may yield insights into vitellogenin receptor biology that may be indicative of the ancestral state in teleost fish. Indeed, scarce information on Lr/Lrps is available from anguillid eels, including putative Vtgrs, to the extent that the Lrp13 member is not yet described. In addition, studies done on the SFE (Damsteegt et al. 2015a; Nguyen et al. 2020) and the European eel (*A. anguilla*: Jéhannet et al. 2019; Morini et al. 2020) did not detect specific transcript variants for *lr8*, as the qPCR primers used were not adequate to differentially amplify the *lr8* + or *lr8-* variants. Consequently, a de novo transcriptome of SFE ovarian tissue was interrogated to examine the LDLr family members expressed in the SFE ovary during early development, with a special interest in putative Vtgrs. Two Lr8 variants, Lr8 + and Lr8-, and an Lrp13 member were found and further characterised.

## Materials and methods

### Sequence analysis

Novel transcript and protein sequences were retrieved from a de novo transcriptome of SFE ovarian tissue in the pre-vitellogenic stage (PV, previous to Vtg incorporation) and the early vitellogenic stage (EV, right after Vtg uptake has started). The database, presented in an earlier publication (i.e., Babio et al. 2022), was interrogated to find the Lr/Lrp members expressed in the SFE ovary. Through the stand-alone command-line application BLAST + package v2.9.0 + provided by NCBI (Camacho et al. 2009), a BLASTp search was done using Lr and Lrp amino acid sequences from other teleost fish (see species names and accession numbers in Online Resource 1). Also, SFE Lr/Lrps were searched by name in the annotated transcriptome. Then, all the associated genes that passed the filter, used to remove lowly expressed genes (Babio et al. 2022), were kept (see sequences excluded from analysis in Online Resource 1). When multiple gene IDs were annotated to the same Lr/Lrp member, only one gene ID was selected based on its highest read counts, unless it was confirmed that they corresponded to different gene sequences after nucleotide and protein alignments.

Subsequently, the corresponding protein sequences from all selected Lr/Lrp members were retrieved. The protein sequences were then subjected to BLASTp search against the nr protein database from NCBI (NCBI Resource Coordinators 2018), and gene/protein nomenclature was based on their corresponding European eel top hit (*A. anguilla* genome RefSeq GCF_013347855.1, Annotation Release 100, accessed in October 2021). For ease of reporting, the putative Vtgrs will be referred to as the *lr8* gene encoding Lr8 +/- variants and *lrp13*/Lrp13, while the genes annotated to Lrp10 and SorLA will be referred to as *lrp10*/Lrp10 and *lr11-like*/Lr11-like, respectively. Protein sequences were further examined, and their conserved domains were identified using the CD-Search tool (Marchler-Bauer and Bryant 2004) from the Conserved Domain Database of NCBI (Lu et al. 2020) and the Simple Modular Architecture Research Tool (SMART: Letunic et al. 2021). Protein sequences were also subjected to phylogenetic analysis (“Phylogenetic analysis” section).

The nucleotide sequences of the putative Vtgrs from SFE were also retrieved, and their open reading frames (ORFs) were detected with the ORF finder from NCBI (available online at https://www.ncbi.nlm.nih.gov/orffinder/, accessed in August 2021). Then, their protein molecular weight was predicted using the Compute pi/MW Tool from the ExPASy server (Gasteiger et al. 2005), and signal peptides were predicted with SignalP v5.0 (Almagro Armenteros et al. 2019). Multiple protein alignments with putative orthologues from different taxa (see species names and accession numbers in Online Resources 2 and 3) were performed using Clustal Omega v1.2.4 (Madeira et al. 2019; Sievers et al. 2011).

### Phylogenetic analysis

**Fig. 1:**
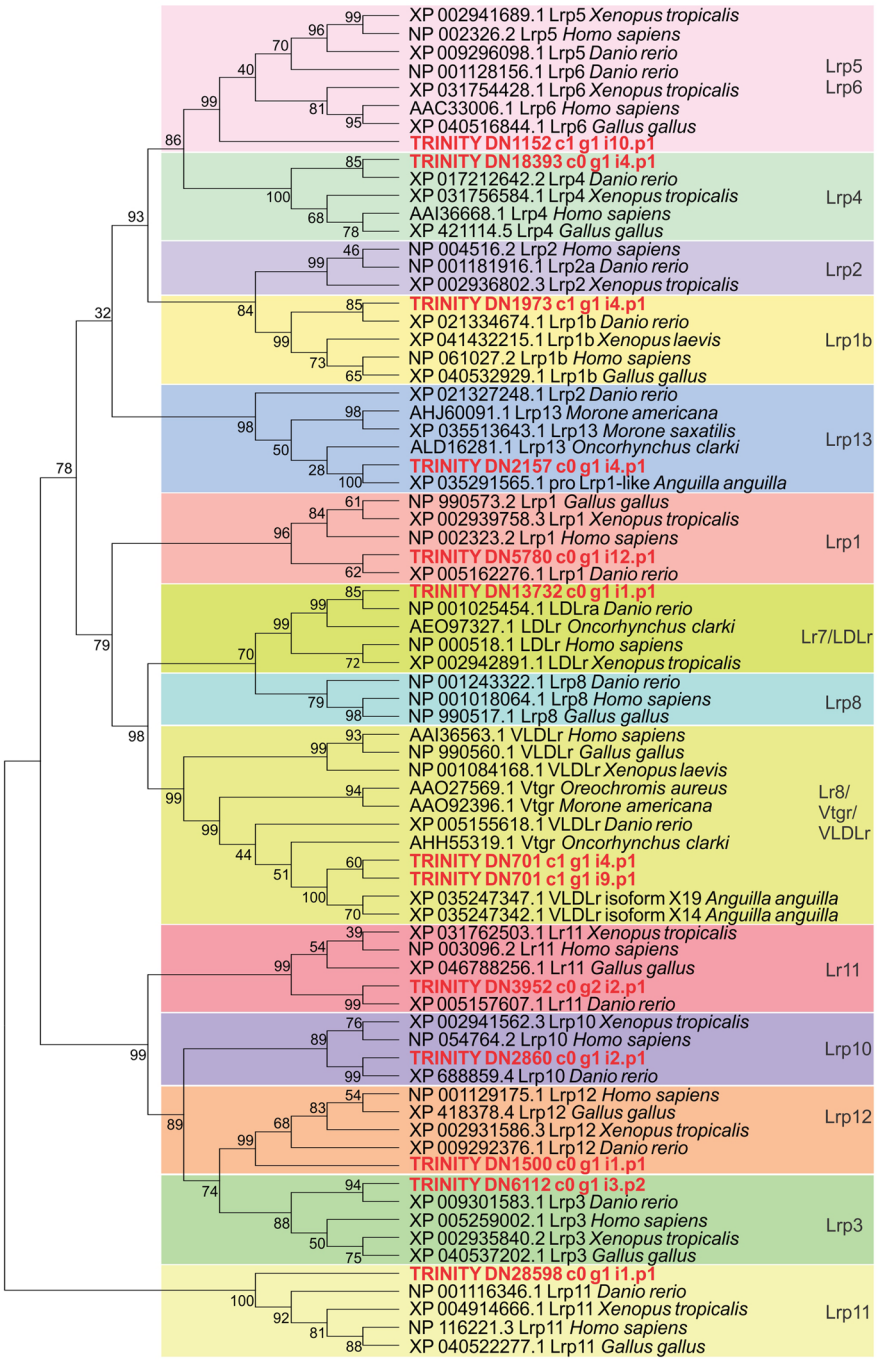
Phylogenetic tree of Lr/Lrp members of the LDLr family from different taxa (accession numbers and species names are shown), including the members expressed in the ovary of the short-finned eel, *Anguilla australis*, denoted by their TRINITY identification in bold red. The tree was constructed using the maximum likelihood method based on the Jones-Taylor-Thornton (JTT) matrix-based mode with a discrete Gamma distribution (*G* = 4.3344), applying 1000 bootstrap replicates. Bootstrapping values (%) are shown at tree nodes

Protein sequences corresponding to Lr/Lrp members of the LDLr family from species representing mammals, amphibians, sauropsids, and teleost fish were retrieved from NCBI (see species names and accession numbers in Fig. 1) to construct a phylogenetic tree including the Lr/Lrp members found in the SFE ovary. Partial sequences (Lrp1-like, Lrp1b-like, Lrp3, Lrp10, and Lrp11) or complete protein sequences (Lr8, Lrp13, LDLr-like, Lrp4-like, Lrp6, Lrp12-like, and Lr11-like) were used (see Online Resource 1 for lengths of sequences retrieved from transcriptome). Only sequences of more than 300 amino acid residues were used; i.e. Lrp5-like was excluded from the analysis. Using MEGA v7 (Kumar et al. 2016), the sequences were first aligned with the ClustalW algorithm (Thompson et al. 1994), and the best model to describe their substitution pattern was then selected based on the lowest Bayesian information criterion (BIC). Briefly, among 56 different amino acid substitution models tested, the Jones-Taylor-Thornton (JTT) matrix-based model (Jones et al. 1992) was selected to be used with a discrete Gamma distribution (four categories, parameter *G* = 4.3344) to model the evolutionary rate differences among sites. Lastly, a tree was constructed with the maximum likelihood method applying 1000 bootstrap replicates. All positions with less than 95% site coverage were eliminated; thus, a total of 316 positions were kept in the final dataset. For visualisation purposes, the Lrp11 group was used as a relative outgroup, similar to Mushirobira et al. (2015).

### Genomic synteny of putative vitellogenin receptors

Due to the lack of SFE genomic data, the surrounding genomic arrangements of the predicted *lr8* and *lrp13* genes for European eel were examined and compared with other teleost fish, using the genomic context section from Entrez Gene (NCBI’s database for gene-specific information, Maglott et al. 2011). The sequences used from the European eel corresponded to the genes encoding the BLASTp top hit using SFE putative Vtgrs as query sequences, i.e. *A. anguilla* genome RefSeq GCF_013347855.1, Annotation Release 100 (accessed in October 2021). The predicted *lr8* orthologues were compared between the European eel (gene ID 118,212,928), zebrafish (gene ID 393,897), and Nile tilapia (gene ID 100,703,410), while predicted *lrp13* orthologues were compared between the European eel (gene ID 118,237,195), zebrafish (gene ID 562,438), and medaka (*Orzyas latipes*, gene ID 101,159,109).

### Tissue distribution of putative vitellogenin receptors

The transcript abundances of the *lr8* variants (two lr8 transcript variants were found, see “Primer design” and “Sequence analysis and phylogenetics” sections) and *lrp13* were estimated in 18 tissues (i.e. red muscle, white muscle, posterior kidney, gill, ovary, anterior kidney, spleen, anterior gut, posterior gut, thyroid, heart, liver, eye, pituitary, head kidney, forebrain, hindbrain, and midbrain) using qPCR (“PCR and qPCR reactions” section) after RNA extraction and cDNA synthesis (“RNA extraction, DNase treatment, and cDNA synthesis” section). All tissues were collected from three wild-caught female eels in the EV stage (Lake Ellesmere, New Zealand; capture year 2020; gonadosomatic index – GSI = 2.54 ± 0.1%, as described previously in Falahati et al. (2021)).

### Expression of putative vitellogenin receptors during ovarian artificial maturation

The ovarian artificial maturation experiment was carried out by Damsteegt et al. (unpublished data) and Mercuriali et al. (unpublished data). Briefly, thirty-five EV stage eels (capture year 2017) were split between a 1000-L recirculating treatment tank (*n* = 30) and a 200-L recirculating control tank (*n* = 5) both maintained at 30–35 ppt, 16–17 °C, 12/12 light/dark regime. Treatment eels received weekly intramuscular injections of salmon pituitary homogenate (SPH, 10 mg/kg, as in Damsteegt et al. 2020), while control eels received weekly injections of eel Ringer’s solution. Before starting the injections, a group of non-treated eels (time point zero (week 0), *n* = 5) was euthanised with an overdose of benzocaine (0.3 g/L, Sigma-Aldrich) and dissected. Similarly, SPH-treated eels were euthanised every 2 weeks, for a maximum of 10 weeks, to detect the progression of oogenesis (*n* = 4–5 per sampled week). Finally, the Ringer-treated eels were euthanised after 10 weeks of treatment (control group – *C* –, *n* = 5). During a routine water change at week 7, the water quality of the tanks was affected, resulting in deaths and reduced sample sizes for week 8 and week 10 (*n* = 4).

During dissections, total body weight and ovary weight were measured to calculate the GSI. Fragments of ovarian tissue were either flash-frozen for RNA extractions (“RNA extraction, DNase treatment, and cDNA synthesis” section) and downstream analysis (qPCR, “PCR and qPCR reactions” section), fixed in Bouin’s for histological analysis, or kept in eel Ringer’s solution for measurement of oocyte diameter (OD) later that same day. Fixed ovarian fragments were dehydrated and embedded in paraffin, sectioned at 5 μm using a Leica RM2125RT microtome (Leica Biosystems, Nussloch, Germany), and stained with hematoxylin and eosin (Damsteegt et al. 2014; Lokman et al. 2016). Micrographs were captured with an Olympus camera SC100 and an Olympus adaptor U-TVO.5XC-3 attached to an Olympus microscope BX51 (Olympus Corporation, Tokyo, Japan). The OD was measured on ovarian fragments subjected to collagenase digestion (1 mg/ml, Lokman et al. 2015). Once isolated follicles were obtained, photographs were taken using an Olympus stereo microscope SZX2-ILLD attached to an Olympus camera SC100 and an Olympus adaptor U-TVO.5XC-3, and images were analysed using the imaging software Olympus CellSens Standard (Olympus Corporation, Tokyo, Japan). In keeping with Lokman et al. (2007), data from the 50% highest-ranked oocytes were retained to calculate the average OD.

### RNA extraction, DNase treatment, and cDNA synthesis

Frozen samples from the tissue distribution assay and the artificial maturation experiment were subjected to total RNA extraction using TRIzol™ Reagent (Invitrogen, Thermo Fisher Scientific Inc.) following the manufacturer’s instructions. Then, 5 μg of total RNA from each sample was treated with DNase TURBO DNA-free™ kit (Invitrogen, Thermo Fisher Scientific Inc.), of which 500 ng was reverse-transcribed with PrimeScript™ RT reagent kit (Takara Bio Inc., Shiga, Japan) using Oligo(dT) and random hexamer primers. Samples were diluted with Milli-Q water (MQW) to 10 ng/μl to be used in qPCR assays (“PCR and qPCR reactions” section).

The cDNA amplified by PCR for subsequent tenfold serial dilution, to construct qPCR standard curves (“Primer design” section), was obtained from an ovarian fragment of one EV eel (capture year 2019: Babio et al. 2022). This sample was subjected to total RNA extraction using the NucleoSpin RNA kit (Macherey–Nagel, Düren, Germany) following the manufacturer’s instructions. The sample was then reverse-transcribed with SuperScript™ IV (Invitrogen, Thermo Fisher Scientific Inc.) using Oligo(dT) primers and used in PCR assays (“PCR and qPCR reactions” section).

### Primer design

The PCR primers employed to generate templates to use as qPCR standards and the qPCR primers used to quantify the β-actin (*actb*) and elongation factor-1α (*eef1a*) transcripts were validated in previous studies (Table 1). PCR and qPCR primers used to amplify *lr8* variants and *lrp13* were designed with Primer3web software v. 0.4.0 (Koressaar and Remm 2007; Untergasser et al. 2012), using the corresponding retrieved sequences (“Sequence analysis” section). To generate qPCR standard curves, the complete ORF from *lr8* variants and *lrp13* were amplified using specific PCR primers (Table 1). Two *lr8* splice variants were found in the SFE, which were amplified using a common pair of primers and then separated based on size selection after electrophoresis. All PCR amplicons were electrophoresed to verify their correct size and gel-extracted using the NucleoSpin gel and PCR clean-up kit (Macherey–Nagel, Düren, Germany) following the manufacturer’s instructions. Once the identity of the PCR products was verified (Sanger sequencing, Genetic Analysis Services, University of Otago), they were subjected to ten-fold serial dilutions in MQW to construct qPCR standards.

Specific qPCR primer pairs were designed to amplify products between 100 and 200 bp for both *lr8* variants and *lrp13* (Table 1). To detect the expression of the *lr8* + variant, qPCR primers were designed to target the region encoding the putative *O*-linked sugar domain. In contrast, to detect the *lr8-* variant, the reverse primer was designed to target the site of this missing domain, i.e. spanning the corresponding exon boundary obtained after splicing (see Online Resource 4). All qPCR amplicons were electrophoresed, gel-extracted, and sequenced to corroborate their identity, as described above.

**Table 1:** PCR and qPCR primers used to amplify cDNAs of the low-density lipoprotein receptor (LDLr) relative with eight ligand-binding repeats (*lr8*), LDLr-related protein-13 (*lrp13*), β-actin (*actb*), and elongation factor-1α (*eef1a*) from short-finned eel, *Anguilla australis*. Amplicon sizes (bp) and annealing temperature (°C) are shown. PCR primers for *lr8* variants and *lrp13* were designed to amplify the complete open reading frames. Lokman PM, George KAN, Divers SL, Algie M, Young G (2007) 11-ketotestosterone and IGF-I increase the size of previtellogenic oocytes from short-finned eel, *Anguilla australis*, in vitro. Reproduction 133:955–967. Setiawan AN, Lokman PM (2010) The use of reference gene selection programs to study the silvering transformation in a freshwater eel *Anguilla australis*: a cautionary tale. BMC Mol Biol 11*:*1471–2199

Target	PCR primers (5′-3′)	Amplicon size (bp)	Ta (°C)	Reference
*lr8*	FW: TATAGCCTACCACGAAATGGTC RV: TGATGTATTGAGAAGGGTAGGG	*lr8* + : 2738 *lr8-*: 2633	52	This study
*lrp13*	FW: CACAACTTTATCGGCGGTCA RV: GAACTTCAGTCTACAGGGGAGGTAA	3734	55	This study
*actb*	FW: AGAGCTACGAGCTGCCTGAC RV: CGGGTGGGGCAATAATCT	561	55	Setiawan and Lokman (2010)
*eef1a*	FW: AAGCAGCTCATTGTGGGAGT RV: AACATTGTCACCGGGAAGAG	703	55	Lokman et al. (2007)
Target	qPCR primers (5′-3′)	Amplicon size (bp)	Ta (°C)	Reference
*lr8* +	FW: TACGGAGCCCTCAAAGAATG RV: CCCTCAGCAGTGACTGGACT	102	61	This study
*lr8-*	FW: GGAGATAATGGCGGCTGTG RV: ACGTTCCCCTCTGAAGGAGG	197	60	This study
*lrp13*	FW: GATCCGACTCGATGGTTCTG RV: AACTGACCACTTCCGTCTTCAC	182	61	This study
*actb*	FW: AATCCTGCGGTATCCATGAG RV: GCCAGGGATGTGATCTCTTT	154	62	Setiawan and Lokman (2010)
*eef1a*	FW: CCCCTGCAGGATGTCTACAA RV: AGGGACTCATGGTGCATTTC	152	62	Setiawan and Lokman (2010)

### PCR and qPCR reactions

All PCR reactions were carried out on an Eppendorf Mastercycler PCR machine. To amplify ORFs encoded by *lr8* variants and *lrp13,* the MyFi Mix (Bioline, Meridina Bioscience, London, UK) was used. Initial denaturation at 95 °C for 2 min was followed by 40 cycles of denaturation at 95 °C for 30 s, annealing for 30 s (see Table 1 for primer-specific annealing temperatures), and extension at 72 °C for 2 min (*lr8* variants) or 2.7 min (*lrp13*). Afterwards, a final extension at 72 °C for 5 min was done. The *actb* and *eef1a* PCR amplifications were done using the MangoTaq™ DNA polymerase kit (Bioline, Meridina Bioscience, London, UK). In this case, the cycling conditions involved an initial denaturation step at 95 °C for 5 min, and 35 cycles of 95 °C for 30 s, annealing for 30 s (see Table 1 for primer-specific annealing temperatures), and extension at 72 °C for 1 min. Subsequently, a final extension at 72 °C for 5 min was done.

All qPCR reactions were carried out on a QuantStudio™ 5 (Applied Biosystems, Thermo Fisher Scientific Inc.), and the results were analysed using QuantStudio™ Design and Analysis Software (Applied Biosystems, Thermo Fisher Scientific Inc.). The reactions were prepared with SYBR® Premix Ex Taq™ II (Takara Bio, Kyoto, Japan) following the manufacturer’s instructions. The cycling conditions started with a hold step at 95 °C for 2 min, followed by 40 cycles of denaturation at 95 °C for 5 s, annealing for 10 s (see Table 1 for primer-specific annealing temperatures), and extension at 72 °C for 5 s. Finally, a melt curve analysis was carried out (95 °C for 1 s, 60 °C for 20 s, and 95 °C for 1 s). Samples were run in duplicate, and where possible all samples, no-template controls and standards were run on the same 96-well qPCR plate. Due to insufficient space, samples from the tissue distribution assay were divided between two plates and run with inter-assay quality controls (two sets of samples amplified on both plates) to determine the coefficient of variation (CV) between runs. Assay efficiencies for the tissue distribution assay were between 97.9 and 102.2% with a CV of 6.4% (*lr8-*), between 92.2 and 93.7% with a CV of 16.0% (*lr8* +), and between 95.1 and 95.2% with a CV of 12.5% (*lrp13*). Assay efficiencies for the artificial maturation experiment were 95.0% (*lr8-*), 95.2% (*lr8* +), 96.7% (*lrp13*), 95.3% (*actb*), and 95.1% (*eef1a*).

### Normalisation of qPCR data

The tissue distribution data were normalised over total RNA. Likewise, after unsuccessful attempts to find suitable reference genes to normalise the expression of target genes during the progression of oocyte development (ovarian artificial maturation experiment), the data were normalised over total RNA. The genes *actb* and *eef1a* were tested and unstable expression across the different developmental stages prevented their use as reference genes (Online Resource 5). In addition, the qPCR data from the ovarian artificial maturation experiment were also analysed with the Markov chain Monte Carlo approach (Matz et al. 2013), showing similar results when compared to the data normalised over total RNA (data not shown). Therefore, the data shown are the normalised over total RNA. The total RNA concentration following DNase treatment was measured using Qubit™ RNA broad range assay kit (Invitrogen, Thermo Fisher Scientific Inc.).

### Statistical analysis

Data are presented as mean ± SEM. Statistical analyses were only performed on the qPCR data obtained from the artificial maturation experiment. Data were tested for normality and homoscedasticity using the Shapiro–Wilk test modified by Rahman and Govindarajulu (1997) and the Levene’s test (Levene 1960), respectively. When assumptions were violated, data were either log-transformed (*lr8-* and *actb* transcript abundances normalised over total RNA, and *lr8-* transcript abundance normalised over *actb*) or analysed with non-parametric tests (*eef1a* transcript abundance normalised over total RNA). Except for this last dataset, each variable was tested using a one-way ANOVA, followed by the Scheffé post-hoc test (Scheffé 1953) due to unequal sample sizes, comparing weeks 0, 2, 4, 6, 8, and 10. Additionally, week 0 and control group C were compared with independent *t*-tests using an adjusted *p* value using the Bonferroni correction. Kruskal Wallis, comparing weeks 0, 2, 4, 6, 8, and 10, or Mann–Whitney *U* test, comparing week 0 and control group C, were used to analyse *eef1a* transcript abundance normalised over total RNA. All analyses were done using InfoStat v.2018 (Di Rienzo et al. 2018).

## Results

### Sequence analysis and phylogenetics

Based on the searches of the transcriptome database (Online Resource 1), the genes encoding the proteins Lr8, Lrp13, LDLr-like, Lrp1-like, Lrp1b-like, Lrp3, Lrp4-like, Lrp5-like, Lrp6, Lrp10, Lrp11, Lrp12-like, and Lr11-like were expressed in the ovary of the SFE during early development (PV and EV stages) (Table 2). The analysis of conserved domains from the deduced protein sequences, along with their alignment with corresponding LDLr family members from different taxa, supports their identity and classification (data only shown for Lr8 and Lrp13 in Online Resources 2 and 3, respectively). Additionally, these members grouped into distinct clusters with their counterparts from different taxa when a phylogenetic tree was constructed, except for Lrp6 which grouped with both Lrp6 and Lrp5 members (Fig. 1).

**Table 2:** Genes encoding Lr/Lrp members of the LDLr family expressed in the ovary of the short-finned eel, *Anguilla australis*, during early development (PV, pre-vitellogenic stage and EV, early vitellogenic stage). The functional annotation of the genes retrieved from the ovarian transcriptome and the top BLASTp hit using their deduced protein sequences as queries are shown. Gene read counts are indicated as mean ± SEM (log_2_ counts per million – logCPM) per stage (n = 6). The protein nomenclature used for each Lr/Lrp is shown. Details of the transcriptome database used have been presented in our earlier publication (Babio L, Lokman PM, Damsteegt EL, Dutoit L (2022) Are cell junctions implicated in the regulation of vitellogenin uptake? insights from an RNAseq-based study in eel, *Anguilla australis*. Cells 11:550)

Nomenclature	Gene ID TRINITY_	Annotation	Top BLASTp hit *A. anguilla*/gene associated	PV reads	EV reads
Lr8 +	DN701_c1_g1_i4	VLDLr [*H. sapiens*]	VLDLr isoform X14 [XP_035247342.1]/*vldlr*	77,093.9 ± 3277.1	74,027.9 ± 2270.2
Lr8-	DN701_c1_g1_i9	VLDLr [*H. sapiens*]	VLDLr isoform X19 [XP_035247347.1]/*vldlr*		
^a^Lrp13	DN2157_c0_g1	Lrp4 [*R. norvegicus*]	proLrp1-like isoform X1 [XP_035291565.1]/LOC118237195	25,991.2 ± 681.5	24,698.5 ± 492.6
Ldlr-like	DN13732_c0_g1	LDLr [*H. sapiens*]	LDLr-like isoform X1 [XP_035254684.1]/LOC118217039	61.3 ± 11.7	161.7 ± 10.0
Lrp1-like	DN5780_c0_g1	Lrp1 [*G. gallus*]	Lrp1-like isoform X2 [XP_035237469.1]/LOC118207712	268.8 ± 74.4	356.5 ± 24.2
Lrp1b-like	DN1973_c1_g1	Lrp1b [*H. sapiens*]	Lrp1b-like isoform X2 [XP_035250378.1]/LOC118214493	491.5 ± 40.5	510.3 ± 46.0
Lrp3	DN6112_c0_g1	Lrp3 [*R. norvegicus*]	Lrp3 [XP_035252614.1]/l*rp3*	79.6 ± 11.7	90.2 ± 15.6
Lrp4-like	DN18393_c0_g1	Lrp4 [*H. sapiens*]	Lrp4-like isoform X2 [XP_035274956.1]/LOC118227957	234.7 ± 17.4	365.3 ± 20.4
Lrp5-like	DN26676_c0_g1	Lrp6 [*H. sapiens*]	Lrp5-like [XP_035252384.1]/LOC118215596	21.7 ± 2.8	30.2 ± 3.3
Lrp6	DN1152_c1_g1	Lrp6 [*H. sapiens*]	Lrp6 [XP_035257773.1]/l*rp6*	1024.8 ± 134.3	1769.5 ± 69.7
Lrp10	DN2860_c0_g1	Lrp10 [*M. musculus*]	hypothetical protein [KAG5846237.1/ANANG_G00147670]	413.9 ± 76.4	860.6 ± 37.5
Lrp11	DN28598_c0_g1	Lrp11 [*H. sapiens*]	Lrp11 [XP_035278838.1]/l*rp11*	18.8 ± 12.3	12.3 ± 1.7
Lrp12-like	DN1500_c0_g1	Lrp12 [*M. musculus*]	Lrp12-like [XP_035288757.1]/LOC118235461	384.0 ± 94.3	1779.9 ± 107.3
Lr11-like	DN3952_c0_g2	SorLA [*O. cuniculus*]	SorLA-like [XP_035288047.1/LOC118235119]	3611.2 ± 480.5	3835.2 ± 566.1

^a^The gene TRINITY_DN2157_c0_g1, functionally annotated as Lrp4, was designated as Lrp13 based on protein sequence analysis (Online Resource 3) and phylogenetic analysis (Fig. 1)

Two genes encoding putative Vtgrs (Lr8 and Lrp13) were found, and their nucleotide and protein sequences were further examined. Based on sequence similarity to other vertebrate Vtgrs, the gene TRINITY_DN701_c1_g1 was designated as *lr8*; two splice variants (TRINITY_DN701_c1_g1_i4 and TRINITY_DN701_c1_g1_i9) of this gene were detected and designated as Lr8 + and Lr8- isoforms, depending on the presence or absence, respectively, of a putative *O*-linked sugar domain (Online Resource 2). The *lr8* + and *lr8-* variants display a 5′-untranslated region (UTR) of 78 bp; ORFs of 2700 bp and 2595 bp, respectively; and a 3′-UTR of 399 bp. The corresponding Lr8 + and Lr8- deduced protein sequences have a length of 899 and 864 amino acids with predicted molecular weights of 99.04 kDa and 95.36 kDa, respectively. Similarly, even though it was functionally annotated as an Lrp4 member (Table 2), the gene TRINITY_DN2157_c0_g1 was designated as *lrp13* due to its structural domains, i.e. seven LDLa repeats at the N-terminal and a unique C-terminal domain containing an extra LDLa repeat (Online Resource 3). The *lrp13* sequence contains a 5′-UTR of 51 bp, an ORF of 3705 bp, and a 3′-UTR of 83 bp, encoding a protein of 1234 amino acids with a predicted molecular weight of 132.76 kDa.

The most likely orthologues of the SFE *lr8* and *lrp13* genes found among the predicted sequences of the European eel (Table 2) were used to examine and compare their syntenic arrangements with other teleost species (see “Genomic synteny of putative vitellogenin receptors” section). The proteins encoded by both genes in both species (SFE *lr8* versus European eel *vldlr* (gene ID 118,212,928) and SFE *lrp13* versus European eel LOC118237195) share a high percentage of identity, supporting their inferred orthology. While SFE Lr8 + and European eel VLDLr isoform X14 share 99.4%, SFE Lr8- and European eel VLDLr isoform X19 share 99.5%. SFE Lrp13 and European eel proLrp1-like share 95.9%.

### Genomic synteny of putative vitellogenin receptors

**Fig. 2:**
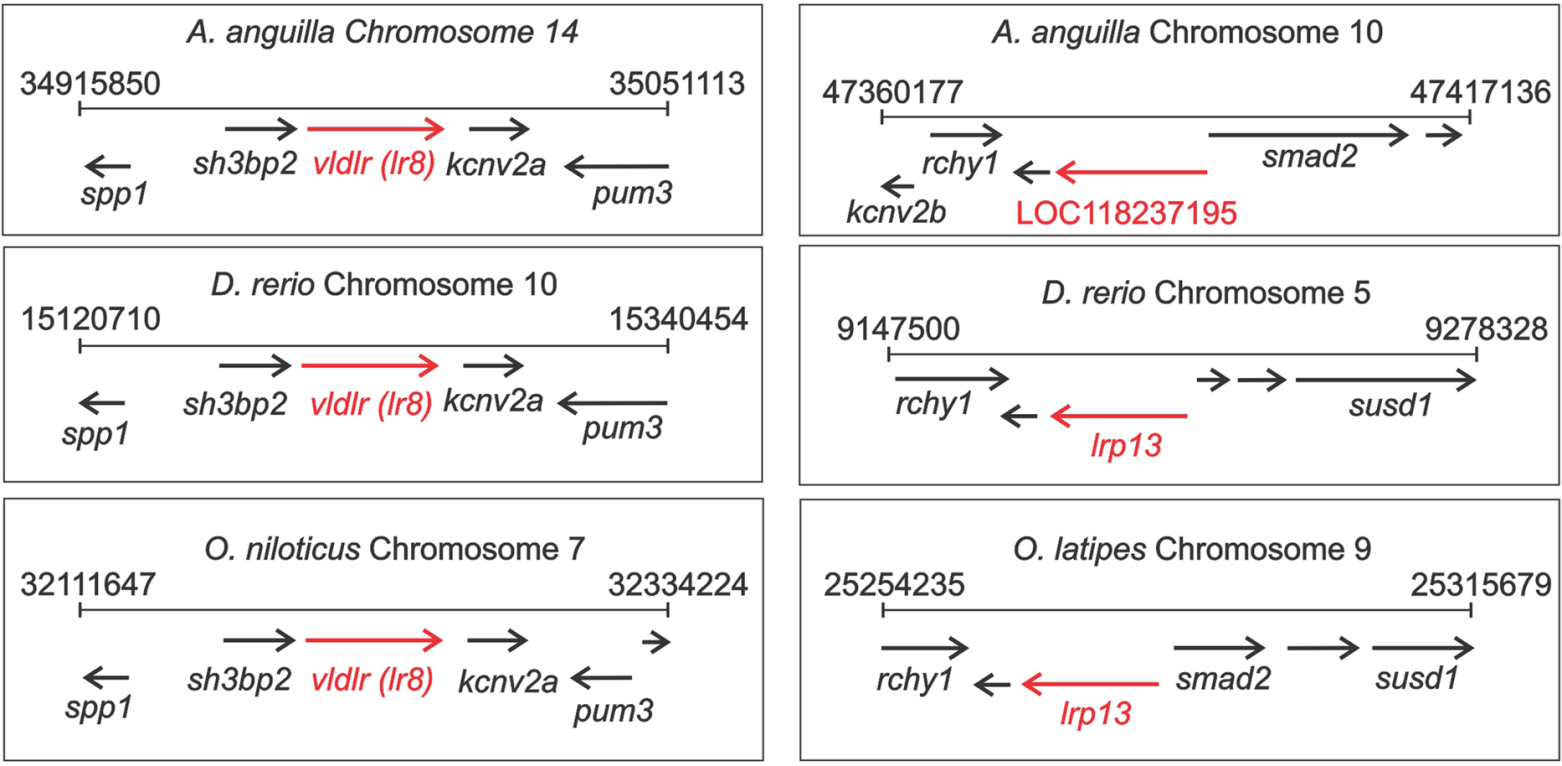
Genomic context of *lr8* and *lrp13* genes in different teleost fish based on the information derived from NCBI database. The European eel (*A. anguilla*), zebrafish (*D. rerio*), medaka (*O. latipes*), and Nile tilapia (*O. niloticus*) were compared. The chromosome number and the regions analysed are indicated for each species. Genes are represented by arrows and named when appropriate. *lr8* and* lrp13* genes are represented by red arrows. *spp1*, secreted phosphoprotein 1; *sh3bp2*, SH3-domain-binding protein 2; *vldlr*/*lr8*; *kcnv2a*, potassium channel subfamily V member 2a; *pum3*, pumilio RNA-binding family member 3; *rchy1*, ring finger and CHY zinc finger domain containing 1; *lrp13*/LOC118237195/*vldlr-like/proLrp1-like*; *smad2*, SMAD family member 2; *susd1*, sushi domain containing 1; *kcnv2b*, potassium channel subfamily V member 2b

Both the *lr8* and *lrp13* genes share genomic synteny when compared with corresponding orthologues from different teleost fish (Fig. 2). Using the NCBI database, the predicted *lr8* (*vldlr*) and *lrp13* (LOC118237195) genomic arrangements for European eel were compared between zebrafish and Nile tilapia and zebrafish and medaka, respectively. For both genes, similar arrangements have been found in the species compared, showing with few exceptions, a retention of direction of the reading frames and the same adjacent genes. Most importantly, the SFE *lr8* and *lrp13* counterparts in the European eel showed similar conserved syntenic arrangements as their orthologues in other teleost fish. In all species compared, the *lr8* gene is consistently surrounded by the genes secreted phosphoprotein 1 (*spp1*) and SH3-domain-binding protein 2 (*sh3bp2*) (upstream) and potassium channel subfamily V member 2a (*kcnv2a*) and pumilio RNA-binding family member 3 (*pum3*) (downstream). The adjacent genes downstream of *lrp13* are slightly more variable between the species compared, but the upstream gene ring finger and CHY zinc finger domain containing 1 (*rchy1*) is present in each species.

### Tissue distribution of putative vitellogenin receptors

**Fig. 3:**
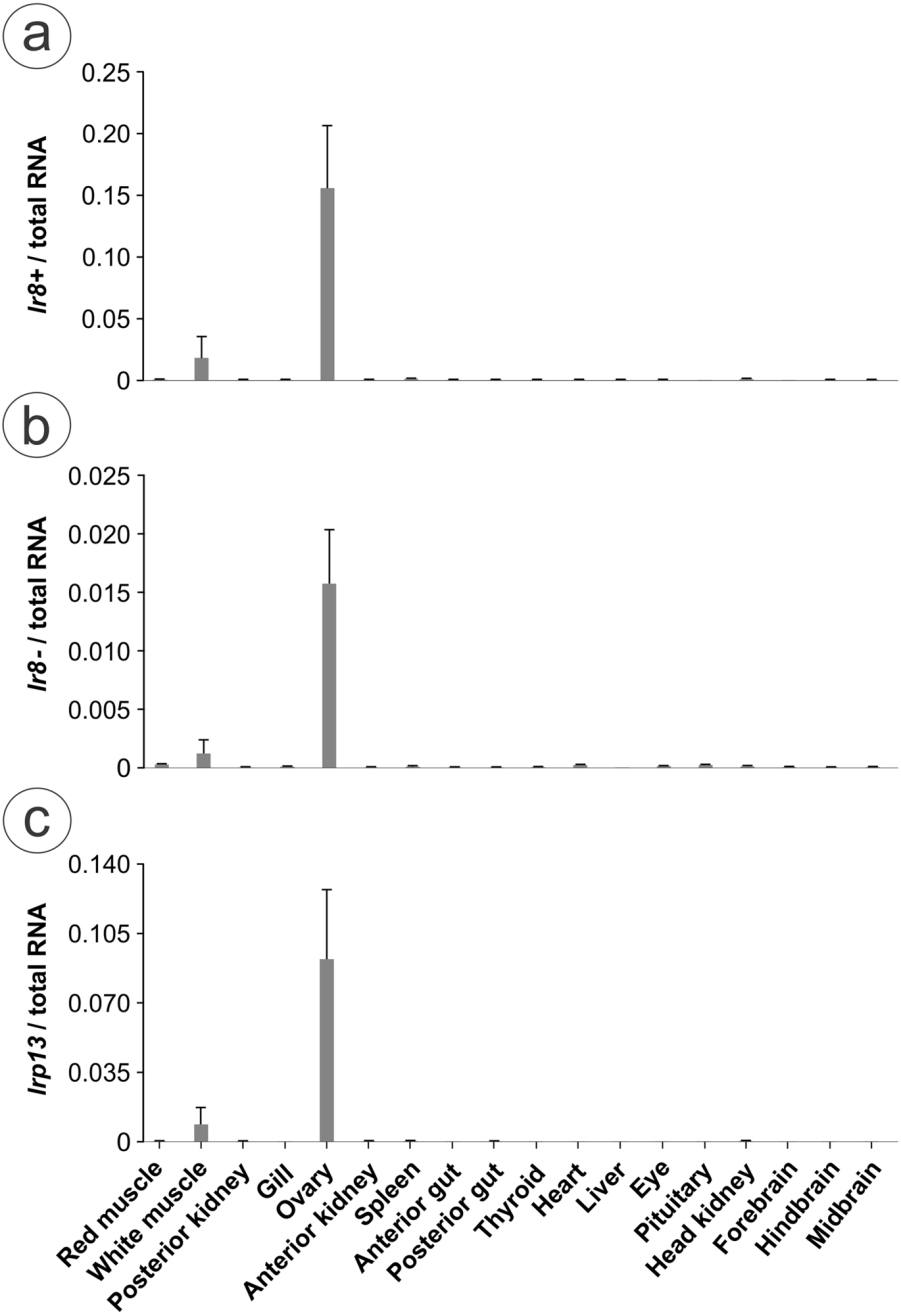
Relative transcript abundance of **a** *lr8* + , **b** *lr8-*, and **c** *lrp13* genes in 18 tissues from the short-finned eel, *Anguilla australis*, during the early vitellogenic stage (*n* = 3). The data, normalised over total RNA, are presented as the mean ± SEM

All putative Vtgrs, i.e. *lr8* + , *lr8-*, and *lrp13*, were highly expressed in the SFE ovary during the EV stage when compared with 17 somatic tissues (Fig. 3). The three transcripts also showed a low expression in white muscle.

### Expression of putative vitellogenin receptor genes during artificial maturation

**Fig. 4:**
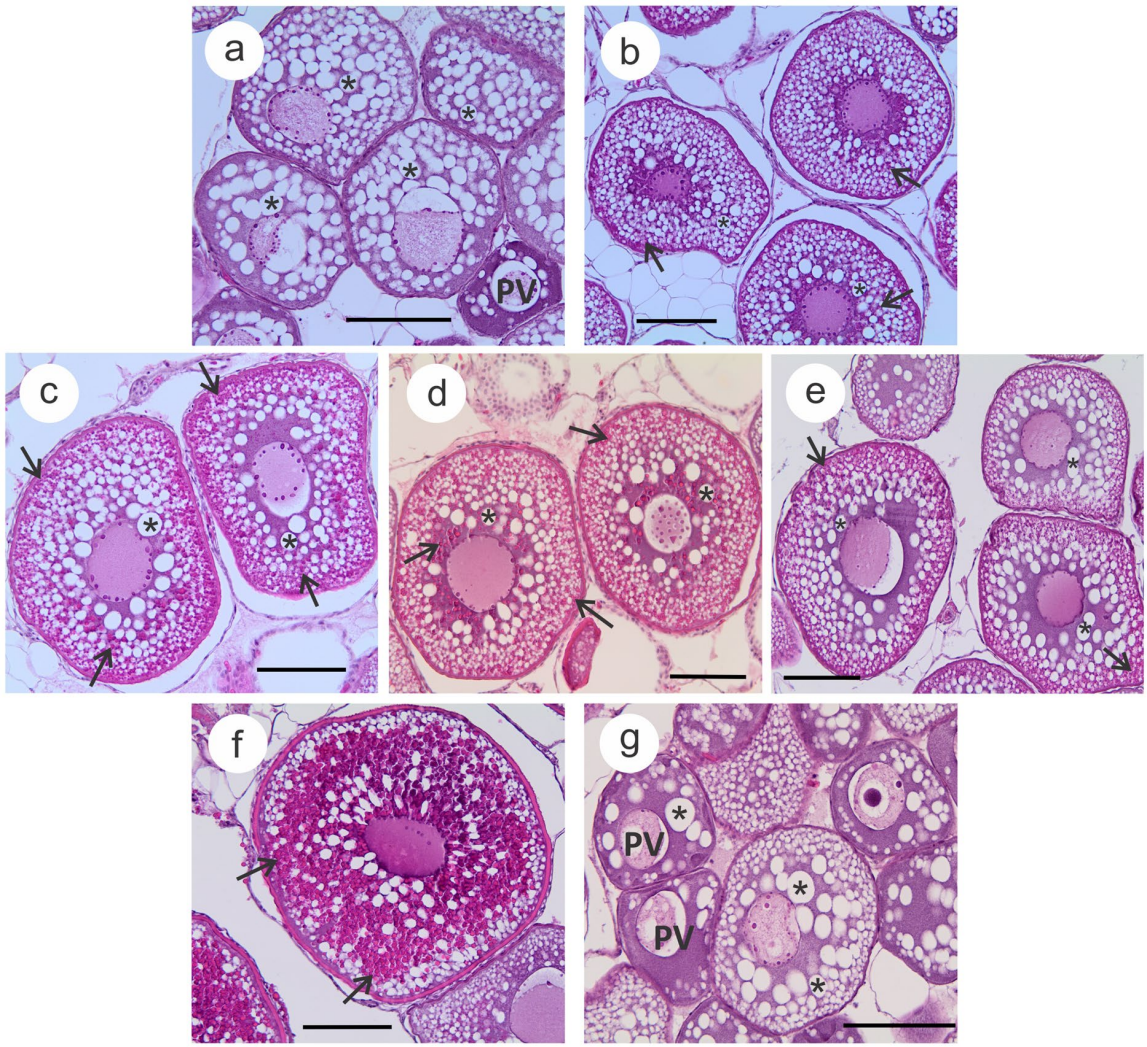
Micrographs of ovaries from short-finned eel, *Anguilla australis*, during artificial maturation stimulated by weekly injections of salmon pituitary homogenate (10 mg/kg) at **b** week 2, **c** week 4, **d** week 6, **e** week 8, or **f** week 10. **a** Non-treated eels at week 0 and **g** eels injected weekly with Ringer’s solution for 10 weeks were used as control groups. The 100 μm scale bars are shown. * indicate oil droplets, arrows indicate yolk proteins, and PV denotes pre-vitellogenic oocytes

The oocyte development during artificial maturation was examined through ovarian histological sections (Fig. 4). During SPH treatment, oocytes started to actively accumulate great amounts of yolk proteins (Vtg accrual), which directed the progress from the EV stage to the late vitellogenic (LV) stage (GSI and OD for week 2 = 4.8 ± 0.5% and 268.2 ± 10.8 μm; week 4 = 6.2 ± 0.8% and 341.2 ± 29.3 μm; week 6 = 11.9 ± 1.5% and 446.7 ± 16.6 μm; week 8 = 6.4 ± 1.3% and 386.9 ± 16.5 μm; week 10 = 23.2 ± 6.1% and 695.0 ± 75.5 μm). In contrast, ovaries from the control groups (week 0 – GSI = 2.2 ± 0.1% and OD = 221.5 ± 4.3 μm – and C – GSI = 2.0 ± 0.1% and OD = 214.9 ± 6.7 μm) were representative of early development, containing both oocytes in the PV and either the late PV stage or EV stage, although no evident yolk accumulation was noted.

**Fig. 5:**
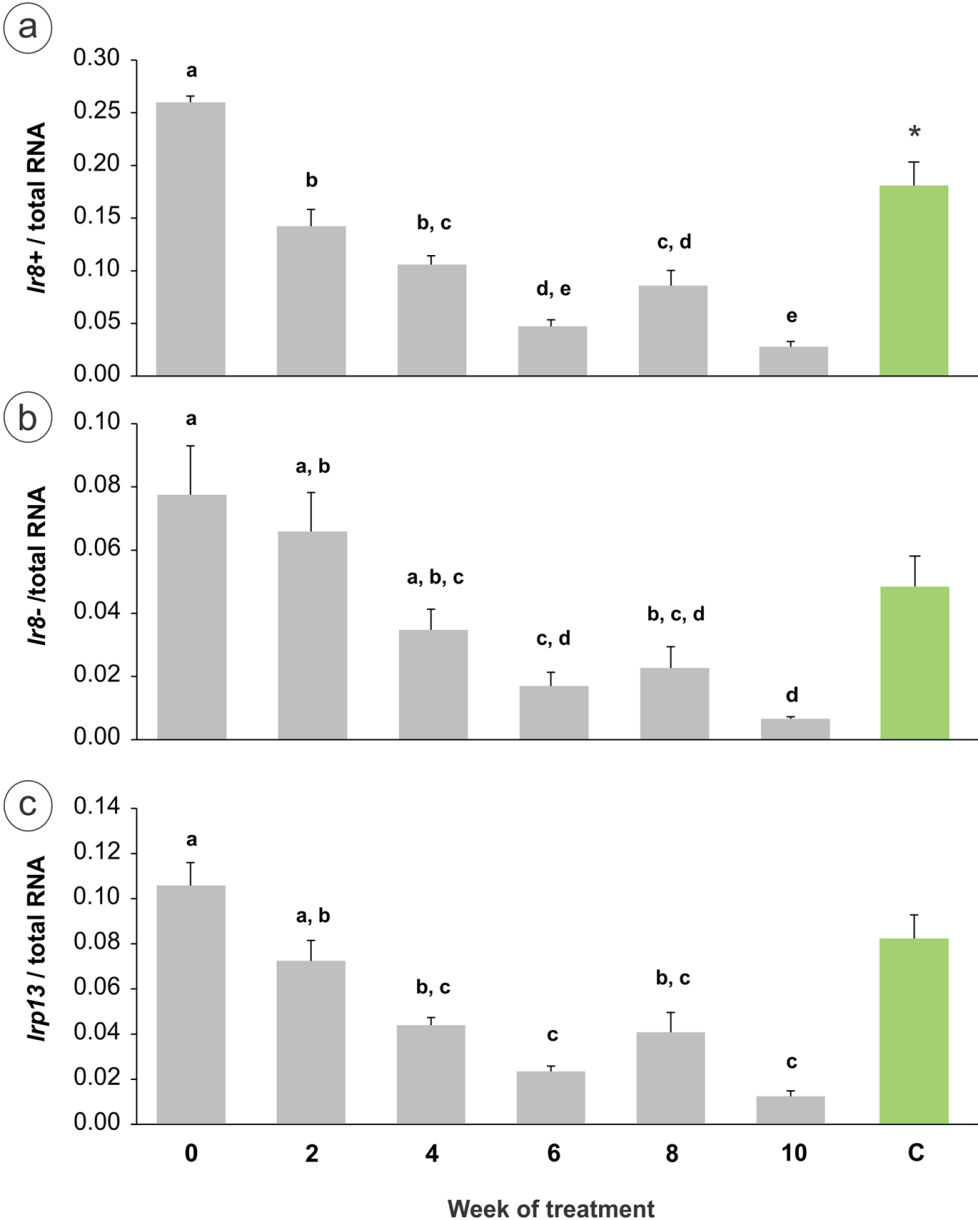
Relative transcript abundance of **a** *lr8* + , **b** *lr8-*, and **c** *lrp13* during induced maturation in the ovary of short-finned eel, *Anguilla australis*. Data are shown as mean ± SEM for females terminally sampled at fortnightly intervals and for Ringer’s-injected control – C – (all groups at *n* = 5, except for weeks 8 and 10 at n = 4). Different letters indicate significant differences between groups (*p* < 0.05; one-way ANOVA: weeks 0, 2, 4, 6, 8, 10). No significant differences were found between week 0 and C groups, except for *lr8* + (*t*-test, * indicates significant difference)

The relative expression of *lr8* + , *lr8-*, and *lrp13* normalised over total RNA decreased throughout progression of oogenesis induced by SPH treatment (one-way ANOVA: *lr8* + : *F* = 68.06, df = 5, *p* < 0.0001; *lr8-*: *F* = 15.31, df = 5, *p* < 0.0001; *lrp13*: *F* = 24.04, df = 5, *p* < 0.0001) (Fig. 5). While no significant differences were found between control groups, i.e. week 0 and C groups, for *lr8-* and *lrp13*, *lr8* + transcript abundance in group C was significantly lower than that at week 0 (*t*-test: *lr8* + ; *t* = 3.43, df = 8, *p* < 0.01). On the contrary, if the data are normalised over *actb*, the relative expression of *lr8* + , *lr8-*, and *lrp13* remains stable during artificial maturation, showing no significant differences in transcript abundance between the experimental groups (*p* > 0.05 for both one-way ANOVA and *t*-test) (see Online Resource 5).

## Discussion

### Lr/Lrp members expressed in ovary during early development

Based on the identification of conserved domains, multiple sequence alignments, and the phylogenetic analysis, several Lr/Lrp members of the LDLr family expressed in the SFE ovary during early development were identified. In addition to the genes encoding putative Vtgrs (Lr8 and Lrp13), the genes encoding Ldlr-like, Lrp1-like, Lrp1b-like, Lrp3, Lrp4-like, Lrp5-like, Lrp6, Lrp10, Lrp11, Lrp12-like, and Lr11-like were found. Since disparate levels of gene expression were detected among them, in comparison to the highly expressed putative Vtgrs (based on the read counts obtained from the transcriptome database, see Table 2), additional research will be needed to elucidate the biological relevance of their expression in the ovary of anguillid eels during early ovarian development, and the functions they may play in teleost fish in general. After phylogenetic analysis, the SFE Lrp6 grouped with both Lrp5 and Lrp6 members from different taxa, likely due to their high homology (Ren et al. 2021). Even though complete Lr/Lrp protein sequences were found for some (i.e. Lr8, Lrp13, LDLr-like, Lrp4-like, Lrp6, Lrp12-like, and Lr11-like), others presented with partial protein sequences (i.e. Lrp1-like, Lrp1b-like, Lrp3, Lrp 5-like, Lrp10, and Lrp11). This possibly affected the phylogenetic analysis, since after alignment, all positions with less than 95% site coverage were eliminated, and a total of 316 residues were considered for the analysis. As putative Lrp5-like, containing only 146 amino acid residues, was excluded from the phylogenetic analysis, it is essential to obtain its whole sequence so as to align it with other Lr/Lrps and corroborate its identification. Additional research is warranted to clarify this.

Receptors from the LDLr family play diverse functions in lipid metabolism, cell homeostasis, and signal transduction pathways (Dieckman et al. 2010; Li et al. 2001; Schneider and Nimpf 2003), being involved in receptor-mediated endocytosis of various ligands, e.g. lipoproteins, hormones, protease inhibitor complexes, vitamins, extracellular matrix proteins, growth factors, and signalling molecules (Dieckman et al. 2010; May et al. 2007). To date, little is known about the function that many of these receptors may play in teleost fish, especially during ovarian development. Indeed, most of the available data on Lr/Lrp function comes from mammals, with a special focus on development and human pathologies. For instance, Lrp1 is widely expressed in several tissues and can play a variety of physiological functions from lipoprotein metabolism, clearance, and degradation of proteases to signalling pathways and development (Lillis et al. 2008). Lrp1b has been mainly implicated in roles in the cell cycle, cellular growth regulation, and proliferation in the context of cancer (Dieckman et al. 2010), similarly to Lrp11, which has been related to oncogenesis and the stress response (Gan et al. 2020; Wang et al. 2019; Xu et al. 2014). Little is known about the CUB domain-containing members Lrp3, Lrp10, and Lrp12, but they have been primarily related to central nervous system development (Cuchillo-Ibañez et al. 2021; Pohlkamp et al. 2017). Also, the SorLA/Lr11 member has been implicated in Alzheimer’s disease pathogenesis (Dieckman et al. 2010). The widely co-expressed paralogous genes encoding Lrp5 and Lrp6 are components of the Wnt signalling pathway, functioning as co-receptors of Wnt ligands (Ren et al. 2021). In addition, Lrp4 has a role in cellular signal transduction regulating the Wnt signalling pathway and also in interacting with other signalling molecules, affecting bone morphogenesis, tooth development, and neuromuscular junction development (see Dieckman et al. 2010 and references therein). In mammals, the Wnt signalling pathway is known to be involved in embryonic development and ovarian differentiation and development, among other functions (Harwood et al. 2008; Tevosian and Manuylov 2008; Zheng et al. 2006). Correspondingly, in rainbow trout, Nicol and Guiguen (2011) detected the expression of several Wnt pathway genes not only during gonadal differentiation, but also during gametogenesis, suggesting the possible implication of this signalling pathway in teleost fish folliculogenesis and/or oogenesis.

Due to their shared ligand-binding properties, many members of the LDLr family can participate in the endocytic uptake of lipoproteins, playing important roles in lipoprotein metabolism and lipid homeostasis. The recognition of circulating lipoproteins is possible through interaction with apolipoproteins (Apos), the protein components of lipoprotein complexes determining also their assembly, structure, and transport (Jonas and Phillips 2008). While the mammalian LDLr, and likely Lrp6 as well (Go and Mani 2012; Ye et al. 2012), bind both ApoE and ApoB (Esser et al. 1988; Gui et al. 2016), the Lr8, Lrp1, and Lr11 members only bind ApoE (Gui et al. 2016; Taira et al. 2001; Zhao et al. 2018). Whereas ApoE and Vtg have long been suggested to be functional analogues (Steyrer et al. 1990), ApoB and Vtg share structural similarities as they both belong to the large lipid transfer protein (LLTP) superfamily (Babin et al. 1999). Notably, the chicken Lr8 is capable of binding both Vtg and VLDL, an ApoB-containing lipoprotein (Stifani et al. 1990), as well as the mammalian ApoE (Steyrer et al. 1990), and blue tilapia’s Vtg has a similar receptor-binding region to ApoB and ApoE (Li et al. 2003). Consequently, it is possible that various Lr/Lrps may be involved in egg yolk formation during oocyte development due to structural similarities between ligands and receptors, respectively.

In teleost species laying fatty eggs, egg yolk formation relies on the uptake and accumulation of Vtg and neutral lipids harboured within low-density lipoproteins, e.g. supplementation of incubation media with VLDL results in lipid uptake in vitro by ovarian fragments of anguillid eels (Endo et al. 2011; Damsteegt et al. 2015b). To date, it is known that whereas the Vtgrs are implicated in egg yolk formation by assisting in Vtg uptake, LDLr likely contributes to neutral lipid incorporation during early development. Although the exact mechanism is unclear, Damsteegt et al. (2015b) showed that the predicted LDLr may be a major player in fatty acid accumulation in *A. australis*. Of note, the human Lrp6 was shown to also regulate LDLr-mediated LDL uptake in vitro, besides its known function as a co-receptor of Wnt ligands (Go and Mani 2012; Ye et al. 2012). In accordance with this, other Lr/Lrps could conceivably assist the main receptors in lipoprotein binding and/or uptake. Nevertheless, it is still unknown if other LDLr family members with similar binding properties, like Lrp1, Lr11, and Lrp6, are implicated in egg yolk formation in teleost fish. Indeed, future research on the Lr/Lrps expressed in the ovary of teleost fish is warranted to elucidate their functions during oocyte development.

Notably, Damsteegt et al. (2015a) analysed the expression of the predicted SFE LDLr during early oogenesis. However, the current ovarian transcriptome database did not yield evidence for *ldlr* expression and instead only showed the expression of a gene encoding a receptor protein closely related to LDLr, the TRINITY_ DN13732_c0_g1 gene encoding the LDLr-like protein. This member was grouped within the Lr7 cluster due to sequence similarity, although it does not seem to correspond to an orthologue of the LDLr; i.e. it only shares 73.75% identity with the predicted European eel LDLr (accession number XP_035262743.1/associated to the *ldlrb* gene), which is orthologous to the zebrafish and human LDLrs. Instead, it was linked to the LDLr-like protein in European eel (accession number XP_035254684.1/associated to the LOC118217039 gene), sharing 96.25% identity. In addition, the gene TRINITY_DN113818_c0_g1, also annotated as LDLr, was found to share 100% identity with the predicted SFE LDLr partial sequence (Damsteegt et al. 2015a) and 98.68% identity with the predicted European eel LDLr. Yet, this gene was excluded from the analysis as it was filtered out due to a low number of reads. After alignment (data not shown), and using the European eel genomic data from NCBI as a reference, both SFE LDLr-like and LDLr seem to be products of different genes, possibly paralogues. This implies that more than one member from the Lr7 group, i.e. LDLr family member containing seven LDLa repeats in its ligand-binding domain, are expressed in the SFE ovary during early development. Targeted studies should be aimed at elucidating the specifics of each gene/protein and their contribution to oocyte development.

### Characterisation of putative vitellogenin receptors

Two genes encoding LDLr family members involved in Vtg binding in some teleost fish, known as *lr8* and *lrp13*, were confirmed to be expressed in the SFE. Lr8 + and Lr8- protein isoforms generated by alternative splicing of transcripts derived from the *lr8* gene were also identified. Both isoforms contain eight LDLa repeats in the ligand-binding domain and only differ in the presence or absence of a putative *O*-linked sugar domain (Lr8 + and Lr8-, respectively). Similar to rainbow trout, both transcript variants differ in 105 bp encoding 35 amino acids representing a putative *O*-linked sugar domain, which presents low levels of homology and glycosylation when compared to the chicken Lr8 + and the human Lr8 + (Prat et al. 1998). According to the predicted European eel *lr8* gene sequence (*vldlr*, gene ID 118,212,928), this region would be encoded by exons 25 and 26, differing from chicken and human *O*-linked sugar domain-coding regions that only span one exon (Bujo et al. 1995; Magrané et al. 1999). Similarly, the rainbow trout *O*-linked sugar domain appears to be encoded by two exons, implying that it may be a common feature in some teleost fish (cf. rainbow trout predicted *lr8* gene sequence NCBI Gene ID: 100,136,065 and regions covered by Lr8 + and Lr8- isoforms—accession numbers XP_036792567.1 and XP_021478075.2, respectively).

Lrp13 proteins found in teleost fish have a species-specific number of N-terminal ligand-binding repeats (7–13) and a unique C-terminal LDLa repeat (+ 1) (Hiramatsu et al. 2013, 2015). For instance, Lrp13 proteins were found in striped bass (*Morone saxatilis*) and white perch (7 + 1) (Reading et al. 2014), cutthroat trout (13 + 1) (Mushirobira et al. 2015), greater amberjack (*Seriola dumerili*) (10 + 1) (Pousis et al. 2019), and yellow croaker (*Larimichthys crocea*) (7 + 1) (Gao et al. 2020). The Lrp13 member expressed in the SFE presents seven LDLa repeats at the N-terminal region and the typical C-terminal configuration of other known Lrp13 proteins. Additional support for the identification of the putative Vtgrs found in the SFE was obtained when examining the syntenic arrangements of the corresponding coding genes using the European eel reference genome. Both the *lr8* and *lrp13* genes share the same syntenic arrangements as their corresponding predicted orthologues from different teleost fish, similar to what was found by Reading et al. (2014) when comparing the zebrafish and Nile tilapia *lr8* and *lrp13* genes, by Andersen et al. (2017) when comparing *lr8* genes from Atlantic salmon, three-spined stickleback, African coelacanth (*Latimeria chalumnae*), spotted gar (*Lepisosteus oculatus*), and elephant fish (*Callorhinchus milii)*, and by Wang et al. (2017) when comparing *lrp13* genes from Chinese tongue sole, medaka, yellow croaker, and Japanese puffer.

After identification and molecular characterisation, the putative Vtgrs from the SFE were further studied, examining their transcript abundance in different somatic tissues. All three putative Vtgrs were expressed almost exclusively in the ovary when compared to 17 somatic tissues from three EV eels, although presenting low expression in white muscle as well. Low expression of *lr8* and *lrp13* genes in muscle has been previously detected in other teleost fish (*lr8*: Gao et al. 2020; Li et al. 2003; Mizuta et al. 2013; Prat et al. 1998 and *lrp13*: Gao et al. 2020; Mushirobira et al. 2015; Reading et al. 2014). Although it remains unclear what role they may play in this tissue and whether their level of expression is significant for physiological function, these receptors may play a part in lipid metabolism facilitating the energy demands of the tissue.

In teleost fish examined to date, the expression of *lrp13* is predominant in ovary (in yellow croaker: Gao et al. 2020; cutthroat trout: Mushirobira et al. 2015; striped bass: Reading et al. 2014; Chinese tongue sole and medaka: Wang et al. 2017). While *lr8* also shows high expression in ovary, its +/- variants usually show differential expression; i.e. the *lr8-* variant is dominant in the ovary in comparison to the *lr8* + variant (e.g. in Senegalese sole: Agulleiro et al. 2007; Atlantic salmon: Andersen et al. 2017; blue tilapia: Li et al. 2003; cutthroat trout: Mizuta et al. 2013; rainbow trout: Prat et al. 1998). Surprisingly, in the ovary of the SFE, the expression of the *lr8* + variant was higher than the expression of the *lr8-* variant. In every qPCR assay performed (to test primers using cDNA pool from three EV eels, capture year 2019; tissue distribution on three EV eels, capture year 2020; artificial maturation experiment with EV eels, capture year 2017), the *lr8* + variant was consistently detected between 1 and 2 Ct values lower than the *lr8-* variant (data not shown). Notably, higher expression could imply higher protein abundance, and thus, protein abundance data should clarify the significant contribution of each receptor to the final Vtgr pool. Whether this expression pattern found in *A. australis*, a basal teleost, reflects the ancestral state and the predominant expression of *lr8-* in ovary from other teleost fish represents the derived state requires further investigation. Additionally, since the only difference between isoforms is the presence of a putative *O*-linked sugar domain in Lr8 + , which in mammalian receptors are thought to confer cell surface stability protecting against shedding proteases (in Lr8 + : Iijima et al. 1998; Magrané et al. 1999; LDLr: Kozarsky et al. 1988; in Lrp8 + : May et al. 2003; Wasser et al. 2014), it may differentially affect the availability of the receptors in the oocyte membrane during vitellogenic growth. Although there are no data available from teleost fish to support this suggestion, it deserves further examination, especially when considering the difference in the levels of glycosylation between teleost fish and mammalian homologues which may affect the function of the domain.

Lastly, *lr8* + , *lr8-*, and *lrp13* gene expression showed the same decreasing trend during ovarian artificial maturation. The expression of the control group in which fish were injected weekly with eel Ringer’s solution for 10 weeks was likely affected by captivity, as it showed lower and/or similar values than week 0 and week 2, although only significant differences were found in *lr8* + transcript abundance. The decreasing expression pattern of Vtgrs during oocyte development has been reported in other teleost fish (e.g. in Atlantic salmon *lr8*: Andersen et al. 2017; largemouth bass, *Micropterus salmoides lr8-*: Dominguez et al. 2012; yellow croaker *lr8-* and *lrp13*: Gao et al. 2020; cutthroat trout *lr8-*: Mizuta et al. 2013; striped bass *lr8- and lrp13*: Reading et al. 2014). However, it is most likely that the changes in RNA composition during oocyte development affect the relative expression of target genes. Indeed, the expression of target genes could be masked or diluted predominantly as a result of a decrease in the mRNA/total RNA ratio during the later stages of development, in part, due to higher expression of 18S and 28S rRNA (Kroupova et al. 2011). Also, the increase by orders of magnitude of specific transcripts during development, such as mitochondrial cytochrome b (Lokman et al. 2003), and conceivably, all other protein-encoding mitochondrial genes, and the accumulation of maternal transcripts during the early stages of oocyte development (Lubzens et al. 2017) could contribute to this effect. Similarly, the dramatic increase in oocyte size during development reflects an increase in cytoplasmic volume promoting the dilution effect. Taken together, this could explain the decreasing trend of expression of the genes analysed, including that of the normaliser genes *actb* and *eef1a*, when oocyte development advances. When data were standardised over total RNA, the relative expression of target genes decreased as the developmental stage advanced, whereas when using *actb*, their expression remained stable. In any case, previous data from other teleost fish affirm that these expression profiles are commonly seen in other teleost fish as Vtgrs either decrease when development advances (see references above) or remain stable (Morini et al. 2020). Physiologically, both scenarios are reasonable as the Vtgrs are likely recycled back to the membrane surface after endocytosis, without de novo synthesis of Vtgr transcripts during vitellogenic growth.

## Conclusions

Multiple Lr/Lrp members of the LDLr family, including putative Vtgrs, are expressed in the SFE ovary during early development, suggesting they play roles in this tissue. The functional redundancy of Lr/Lrp members (Schneider et al. 1999) may indicate that (some of) these roles can overlap between multiple members, likely contributing to key steps in oocyte development and possibly assisting in egg yolk development. However, the possible functions that the Lr/Lrp members play in supporting egg yolk formation and/or cellular signalling pathways involved in oocyte development need to be further explored in the SFE and teleost fish in general. Additionally, it has to be determined if their level of expression has any biological significance. Finally, three putative Vtgr receptors, i.e. Lr8 + , Lr8-, and Lrp13, known to be involved in Vtg uptake and egg yolk formation in some fish, which ultimately translates into egg quality and reproductive effort, have been characterised in the SFE*.* The three genes are mainly expressed in ovary when compared to somatic tissues, and their expression decreases as oocyte development advances by artificial means. The expression pattern found in the SFE, in which both *lr8* variants are mainly expressed in ovary and *lr8* + expression is higher than *lr8-*, is in contrast to what was detected in other oviparous vertebrates. Further studies in anguillid eels are needed to confirm this pattern and specify the contribution of each variant to the final Vtgr pool available for Vtg uptake.

## Supplementary Information

Below is the link to the electronic supplementary material.

Supplementary file1 (ZIP 631 KB)

## Data Availability

The data presented in this study is contained within the article and supplementary material.
