# Supplementary material for: Lipoprotein receptors in ovary of eel, Anguilla australis: molecular characterisation of putative vitellogenin receptors
Source: Fish Physiol Biochem. 2023 Jan 17;49(1):117–37. doi: 10.1007/s10695-023-01169-6 (PMC9935665; doi:10.1007/s10695-023-01169-6)
Supplement: Supplementary file 1 — Supplementary file1 (ZIP 631 KB) [file 10695_2023_1169_MOESM1_ESM.zip › Online Resource 1.pdf]

Fish Physiology and Biochemistry

"Lipoprotein receptors in ovary of eel, *Anguilla australis*; molecular characterisation of putative vitellogenin receptors"

Lucila Babio*; Erin L. Damsteegt; and P. Mark Lokman.
Department of Zoology, University of Otago, Dunedin, New Zealand.
*Corresponding author (e-mail: lucilababio@gmail.com). Department of Zoology, University of Otago, 340 Great King Street, P.O. Box 56, Dunedin 9054, New Zealand.

Online Resource 1 Lipoprotein receptors and related proteins found in an ovarian transcriptome database of shortfinned eel, *Anguilla australis*. Target sequences were retrieved by BLASTp using protein sequences from other teleost fish as queries. The percent identity along with the queries' accession numbers and species names of each search are shown. The length of the retrieved sequences are also shown (amino acid residues – aa –). Details of the transcriptome database used have been presented in our earlier publication (Babio et al. 2022)

Query Lr/Lrp	Query species	Query accession number	BLASTp top hit result	Percent identity	Length (aa)
Lr8-	*O. clarki*	AHH55319.1	TRINITY_DN701_c1_g1_i9.p1	87.85%	864
Lr8-	*O. clarki*	AHH55319.1	TRINITY_DN701_c1_g1_i4.p1	83.65%	899
Lrp13	*M. americana*	AHJ60091.1	TRINITY_DN2157_c0_g1_i4.p1	51.82%	1234
Lrp13	*O. clarki*	ALD16281.1	TRINITY_DN2157_c0_g1_i4.p1	56.43%	-
LDLr	*O. clarki*	AEO97327.1	TRINITY_DN13732_c0_g1_i1.p1	65.00%	853
LDLr	*A. australis*	AIC83955.1	TRINITY_DN13732_c0_g1_i1.p1	74.55%	-
LDLr	*A. australis*	AIC83955.1	[a]TRINITY_DN113818_c0_g1_i1.p4	100%	-
Lrp1	*D. rerio*	XP_005162276.1	TRINITY_DN5780_c0_g1_i12.p1	79.49%	2226
Lrp1B	*D. rerio*	XP_021334674.1	TRINITY_DN1973_c1_g1_i4.p1	77.29%	2344
Lrp2	*D. rerio*	NP_001181916.1	[a]TRINITY_DN26618_c0_g1_i1.p1	58.26%	-
Lrp3	*D. rerio*	XP_009301583.1	TRINITY_DN6112_c0_g1_i3.p2	70.85%	420
Lrp4	*D. rerio*	XP_017212642.2	TRINITY_DN18393_c0_g1_i4.p1	82.88%	1899
Lrp5	*D. rerio*	XP_009296098.1	TRINITY_DN26676_c0_g1_i1.p3	88.23%	146
Lrp6	*D. rerio*	NP_001128156.1	TRINITY_DN1152_c1_g1_i10.p1	82.96%	1619
Lrp8	*D. rerio*	NP_001243322.1	[a]TRINITY_DN23579_c0_g2_i1.p1	77.49%	-
Lrp10	*D. rerio*	XP_688859.4	TRINITY_DN2860_c0_g1_i2.p1	73.35%	364
Lrp11	*D. rerio*	NP_001116346.1	TRINITY_DN28598_c0_g1_i1.p1	72.45%	326
Lrp12	*D. rerio*	XP_009292376.1	TRINITY_DN1500_c0_g1_i1.p1	76.58%	841
Lr11	*D. rerio*	XP_005157607.1	TRINITY_DN3952_c0_g2_i2.p1	76.48%	2221

[a] The sequences TRINITY_DN113818_c0_g1_i1.p4, TRINITY_DN26618_c0_g1, and TRINITY_DN23579_c0_g2_i1.p1 were excluded from the analysis as they were filtered out due to low read counts (Babio et al. 2022). Babio L, Lokman PM, Damsteegt EL, Dutoit L (2022) Are cell junctions implicated in the regulation of vitellogenin uptake? insights from an RNAseq-based study in eel, *Anguilla australis*. Cells 11:550
