# Supplementary material for: Lipoprotein receptors in ovary of eel, Anguilla australis: molecular characterisation of putative vitellogenin receptors
Source: Fish Physiol Biochem. 2023 Jan 17;49(1):117–37. doi: 10.1007/s10695-023-01169-6 (PMC9935665; doi:10.1007/s10695-023-01169-6)
Supplement: Supplementary file 1 — Supplementary file1 (ZIP 631 KB) [file 10695_2023_1169_MOESM1_ESM.zip › Online Resource 2.pdf]

Fish Physiology and Biochemistry

"Lipoprotein receptors in ovary of eel, *Anguilla australis*; molecular characterisation of putative vitellogenin receptors"

Lucila Babio*; Erin L. Damsteegt; and P. Mark Lokman.

Department of Zoology, University of Otago, Dunedin, New Zealand.

*Corresponding author (e-mail: lucilababio@gmail.com). Department of Zoology, University of Otago, 340 Great King Street, P.O. Box 56, Dunedin 9054, New Zealand.

Online Resource 2 *Anguilla australis* (shortfinned eel, SFE) Lr8+/- isoforms aligned with rainbow trout (CAD10640: Davail et al. 1998), cutthroat trout (AHH55319.1: Mizuta et al. 2013), white perch (AAO92396.1: Hiramatsu et al. 2004), blue tilapia (AAO27569.1: Li et al. 2003), and chicken (NP_990560.1: Bujo et al. 1994) Lr8- isoforms, and human Lr8+ (VLDLr, AAI36563.1: Webb et al. 1994). The signal peptide, the LDLa repeats, the EGF-like repeats, the putative *O*-linked sugar domains, and the transmembrane region are demarcated. The conserved sequences DxSDE, F/YWxD, and FxNPxY are highlighted. Alignment was performed using Clustal Omega v1.2.4. The "*" represents fully conserved residues, ":" indicates residues with strong similar properties, and "." represents residues with weak similar properties

```
Lr8+ SFE             ----------------MVTSILGFLIL--PICLQ-----QCGYVQGTRTECEPSQYQCGNGRCIPSVWQCDGDEDCSDGSDETTCVKKTCAEADFVCNNGQ 78
Lr8- SFE             ----------------MVTSILGFLIL--PICLQ-----QCGYVQGTRTECEPSQYQCGNGRCIPSVWQCDGDEDCSDGSDETTCVKKTCAEADFVCNNGQ 78
Lr8- white perch     ----------------MVTSTPGILLLPMLICLQ-----HCSNVHGTKTECEASQFQCGNGRCIPSVWQCDGDEDCTDGSDENSCVRKTCAEVDFVCRNGQ 80
Lr8- blue tilapia    ----------------MVTSTQGILLLPMLICLQ-----HFVNVHGTKTECEANQFQCGNGRCIPSVWQCDGDEDCSDGSDENSCVKKTCAELDFVCDNGQ 80
Lr8- rainbow trout   ----------------MLTSILEILIL--RICLQ-----QCGFVHGSKTECEPSQFQCGNGRCIPSVWQCDGDEDCSDGSDENTCVRKTCAEVDFVCRNGQ 78
Lr8- cutthroat trout ----------------MLTSILEILIL--RICLQ-----QCGFVHGSKTECEPSQFQCGNGRCIPSVWQCDGDEDCSDGSDENTCVRKTCAEVDFVCRNGQ 78
Lr8- chicken         MRSSRQRGDRSAATGGGCGARRWALPRCGALCLLLALGCLRTATDGAKAKCEESQFQCSNGRCIPLLWKCDGDEDCSDGSDESACVKKTCAESDFVCNSGQ 101
Lr8+ human           ----------------MGTSALWALWLLLALCWA--PRESGATGTGRKAKCEPSQFQCTNGRCITLLWKCDGDEDCVDGSDEKNCVKKTCAESDFVCNNGQ 83
                                        :    *     :*             * :::** .*:** *****  :*:******* *****. **:***** **** .**
                                     <----- SIGNAL PEPTIDE ----->      <-------------- LDLa 1 ----------> <---------------

Lr8+ SFE             CVPKRWHCDGEPDCEDGSDESLEVCHTRTCRVNEFSCGAGTTQCIPVFWKCDRERDCDNGEDEVNCGNITCAPLEFTCASGRCISRNFACNGEDDCGDGSD 179
Lr8- SFE             CVPKRWHCDGEPDCEDGSDESLEVCHTRTCRVNEFSCGAGTTQCIPVFWKCDRERDCDNGEDEVNCGNITCAPLEFTCASGRCISRNFACNGEDDCGDGSD 179
Lr8- white perch     CVPKRWHCDGEPDCEDGSDESVEICHMRTCRVNEFSCGAGSTQCIPIFWKCDGEKDCDNGEDEVHCGNITCAPNEFTCASGRCISRNFVCNGEDDCGDGSD 181
Lr8- blue tilapia    CVPKRWHCDGEPDCEDGSDESLDICHMRTCRMNEFSCGAGSTQCIPVFWKCDGEKDCDNGEDEVNCGNITCAPNEFTCASGRCISRNFVCNGEDDCGDGSD 181
Lr8- rainbow trout   CVPKRWHCDGEPDCEDGSDERVEVCHTRTCRVNEFSCGAGSTQCIPVFWKCDGEKDCDHGEDEMSCGNITCASLEFTCASGRCISLNFVCNGEDDCGDGSD 179
Lr8- cutthroat trout CVPKRWHCDGEPDCEDGSDERVEVCHTRTCRVNEFSCGAGSTQCIPVFWKCDGEKDCDHGEDEMSCGNITCASLEFTCASGRCISLNFVCNGEDDCGDGSD 179
Lr8- chicken         CVPNRWQCDGDPDCEDGSDESAELCHMRTCRVNEISCGPQSTQCIPVSWKCDGEKDCDSGEDEENCGNVTCSAAEFTCSSGQCISKSFVCNGQDDCSDGSD 202
Lr8+ human           CVPSRWKCDGDPDCEDGSDESPEQCHMRTCRIHEISCGAHSTQCIPVSWRCDGENDCDSGEDEENCGNITCSPDEFTCSSGRCISRNFVCNGQDDCSDGSD 184
                     ***.**:***:*********  : ** ****::*:***  :*****: *:** *.*** ****  ***:**:  ****:**:*** .*.***:***.****
                     --- LDLa 2 ------------> <-------------- LDLa 3 ----------------> <------------- LDLa 4 -------------
```

```
Lr8+ SFE               ELDCTPSSCGPSEFQCGNATCIPASWVCDEDVDCQDQSDESPQRCGQRPPTPPTKCPSSETQCGSGECIHRKWRCDGDPDCKDGSDEANCPVRTCRPDQFK 280
Lr8- SFE               ELDCTPSSCGPSEFQCGNATCIPASWVCDEDVDCQDQSDESPQRCGQRPPTPPTKCPSSETQCGSGECIHRKWRCDGDPDCKDGSDEANCPVRTCRPDQFK 280
Lr8- white perch       EVECAPSSCGPSEFQCGNSSCIPASWVCDDDVDCQDQSDESPTRCGRH-PTPPAKCSSSEMQCRSGECIHKKWRCDGDPDCKDSSDEANCPVRTCGPDQFK 281
Lr8- blue tilapia      EVECAPSSCGPSEFQCGNSSCIPASWVCDDDVDCQDQSDESPSHCGRH-PTPPAKCSPSEMQCRSGECIHKKWRCDGDSDCKDGSDEANCPVRTCGSDQFK 281
Lr8- rainbow trout     EQECAPSSCGPSEFQCGNATCIPGNWVCDDDVDCQDQSDESPQRCGRQ-PTPPAKCSSSETQCGSGECIHRKWQCDGDPDCKDGSDEANCSVRTCRPDQFK 279
Lr8- cutthroat trout   EQECAPSSCGPSELQCGNSTCIPGNWVCDDDVDCQDQSDESPQRCGRQ-PTPPAKCSSSETQCGSGECIHRKWRCDGDPDCKDGSDEANCSVRTCRPDQFK 279
Lr8- chicken           ELECAPPTCGVHEFQCKSSTCIPISWVCDDDADCSDHSDESLEQCGRQ-PAPPVKCSTSEVQCGSGECIHKKWRCDGDPDCKDGSDEINCPSRTCRPDQFR 302
Lr8+ human             ELDCAPPTCGAHEFQCSTSSCIPISWVCDDDADCSDQSDESLEQCGRQ-PVIHTKCPASEIQCGSGECIHKKWRCDGDPDCKDGSDEVNCPSRTCRPDQFE 284
                       * :*:* :**  *:** .::*** .****:*.**.*:****  :**:: *.  .**  ** ** ******:**:**** ****.*** **  ***  ***.
                       --> <------------- LDLa 5 ----------------> <---------------------- LDLa 6 ------------> <----------

Lr8+ SFE               CDDGNCIHGSRQCNGLRDCADGSDEMNCKNVTQCNGPDKFKCRSGECIEMSKVCNKNRDCPDWSDEPLRECNLNECLLNNGGCSHICRDMVIGYECDCTPG 381
Lr8- SFE               CDDGNCIHGSRQCNGLRDCADGSDEMNCKNVTQCNGPDKFKCRSGECIEMSKVCNKNRDCPDWSDEPLRECNLNECLLNNGGCSHICRDMVIGYECDCTPG 381
Lr8- white perch       CDDGNCILGSRQCNSHRDCTDGSDEVNCKDMTQCNGPEKFKCRSGECIEMSKVCNKVRDCPDWSDEPIKECNLNECLLNNGGCSHICKDMVIGFECDCTPG 382
Lr8- blue tilapia      CDDGNCILGSRQCNGLRDCADGSDEANCRNMTQCNGPEKFKCRSGECIEMSKVCNKVRDCPDWSDEPIKECNFNECLLNNGGCSHICKDMVIGYECDCTPG 382
Lr8- rainbow trout     CEDGNCIHGSRQCNGLRDCADGTDEVNCKNLTQCNGPDKFKCRSGECIEMSKVCNKARDCPDWSDEPINECNLNECLLNNGGCSHICRDMVIGFECDCTPG 379
Lr8- cutthroat trout   CEDGNCIHGSRQCNGLRDCADGTDEVNCKNLTQCNGPDKFKCRSGECIEMSKVCNKARDCPDWSDEPINECNLNECLLNNGGCSHICRDMVIGFECDCTPG 379
Lr8- chicken           CEDGNCIHGSRQCNGVRDCLDGTDEANCNNVIQCSGPGKFKCRSGECIDINKVCNHHGDCKDWSDEPLKECNINECLVNNGGCSHICRDLVIGYECDCPAG 403
Lr8+ human             CEDGSCIHGSRQCNGIRDCVDGSDEVNCKNVNQCLGPGKFKCRSGECIDISKVCNQEQDCRDWSDEPLKECHINECLVNNGGCSHICKDLVIGYECDCAAG 385
                       *:**.** ******. *** **:** **.:: ** ** **********::.****:  ** ******:.**::****:*********:*:***:****  *
                       ------- LDLa 7 -----------> <---------------- LDLa 8 ------------------>    <------------ EGF A -------

Lr8+ SFE               LQLIDRKTCGDINECLNPGICSQICINLKGGYKCECHSGYQMDPTSGVCKAVGKEPCLIFTNRRDIRKLGLERMEYTQIVEQLRNTVALDADFTQQRIFWA 482
Lr8- SFE               LQLIDRKTCGDINECLNPGICSQICINLKGGYKCECHSGYQMDPTSGVCKAVGKEPCLIFTNRRDIRKLGLERMEYTQIVEQLRNTVALDADFTQQRIFWA 482
Lr8- white perch       LQLIDHKTCGDINECLNPGICSQICINLKGGYKCECHNGYQMDPTTGVCKAVGKEPCLIFTNRRDIRRLGLERKEYTQIVEQQRNTVALDADFNQQMIFWA 483
Lr8- blue tilapia      LQLIDHKTCGDIDECMNPGICSQICINLKGGYKCECHNGDQMDPTTGVCKAVGKEPCLIFTNRRDIRRLGLERKEYTQIVEQQRNAVALDADFDQQTIFWA 483
Lr8- rainbow trout     LQLIDRKTCGDINECMNPGICSQICINLKGGYKCECHNSYQMDPTTGVCKAVGKEPCLIFTNRRDIRKLGLERREYTQIVEQLRNTVALDADFTQQRIFWA 480
Lr8- cutthroat trout   LQLIDRKTCGDINECMNPGICSQICINLKGGYKCECHNSYQMDPTTGVCKAVGKEPCLIFTNRRDIRKLGLERREYTQIVEQLRNTVALDADFTQQRIFWA 480
Lr8- chicken           FELVDRRTCGDIDECQNPGICSQICINLKGGYKCECSRGYQMDLATGVCKAVGKEPCLIFTNRRDIRKIGLERKEYIQLVEQLRNTVALDADIAEQKLYWA 504
Lr8+ human             FELIDRKTCGDIDECQNPGICSQICINLKGGYKCECSRGYQMDLATGVCKAVGKEPSLIFTNRRDIRKIGLERKEYIQLVEQLRNTVALDADIAAQKLFWA 486
                         ::*:*::*****:*********************  . *** ::**********.**********::**** ** *:*** **:******:  * ::**
                       ------->     <-------------- EGF B ------------>

Lr8+ SFE               DLGQKAIFSTVLDKRDEVNSHTKVIDNVQTPVGIAVDWIYKNIYWSDLGTKTITVADFSGAKRKVLFNSGLKEPASIAVDPLSGFLYWSDWGEPAKIEKSG 583
Lr8- SFE               DLGQKAIFSTVLDKRDEVNSHTKVIDNVQTPVGIAVDWIYKNIYWSDLGTKTITVADFSGAKRKVLFNSGLKEPASIAVDPLSGFLYWSDWGEPAKIEKSG 583
Lr8- white perch       DLGQKAIFSTVLDKRVEVGTHNKVINNVQTPVGIAVDWIYKNLYWSDLGTKTISVANFNGTKQKVLFNRGLKEPASIAVDPLSGFLYWSDWGEPAKIEKSG 584
Lr8- blue tilapia      DLGQKAIYSTLLDKRVDVGTHKKVIDNVQTPVGIAVDWIYKNLYWSDLGTKTISVASFNGTKQKVLFNRGLKEPADIAVDPLSGFLYWSDWGEPAKIEKSG 584
Lr8- rainbow trout     DLSQRAIFSTVLDKRGDVGSHVKVIDNV-TPVGIAVDWIYNNIYWSDLGTKTIEVANFNGTKRKVLFSSGLKEPASIAVDPLSGFLYWSDWGEPATIEKSG 580
Lr8- cutthroat trout   DLSQRAIFSTVLDKRGDVGSHVKVIDNV-TPVGIAVDWIYNNIYWSDLGTKTIEVANFNGTKRKVLFSSGLKEPASIAVDPLSGFLYWSDWGEPAKIEKSG 580
Lr8- chicken           DFSQKAIFSASIDTRDKVGTHTRILDNIHSPAGIAVDWIYKNIYWTDSSAKTISVASLNGKKRKVLFLSELREPASIAVDPLSGFMYWSDWGEPAKIEKAG 605
Lr8+ human             DLSQKAIFSASID--DKVGRHVKMIDNVYNPAAIAVDWVYKTIYWTDAASKTISVATLDGTKRKFLFNSDLREPASIAVDPLSGFVYWSDWGEPAKIEKAG 584
                       *:.*:**:*: :*    .*. * ::::*: .*..*****:*:.:**:* .:*** ** :.* *:*.**   *:***.*********:*********.***:*
```

```
Lr8+ SFE             MNGVDRQVLVATDIQWPNGITLDLIKSRLYWVDSKLHMLCSVDLNGDNRRKVLQSQDYLAHPFALTVFEDRVFWTDGENEAIYGANKFTGTDVITLASNLN 684
Lr8- SFE             MNGVDRQVLVATDIQWPNGITLDLIKSRLYWVDSKLHMLCSVDLNGDNRRKVLQSQDYLAHPFALTVFEDRVFWTDGENEAIYGANKFTGTDVITLASNLN 684
Lr8- white perch     MNGVDRQVLVASDIQWPNGITLDLIKGRLYWVDSKLHMLCSVDLNGDNRKKVLQSADYLAHPFALTVFEDRVFWTDGENEAIYCANKFTGSDVVTLASNLN 685
Lr8- blue tilapia    MNGVDRQVLVATDIQWPNGITLDLIKGRLYWVDSKLHMLCSVDLNGDNRNKVLQSSEYLAHPFALTVFEDRVFWTDGEKEAIYGANKFTGSDVVTLASNLN 685
Lr8- rainbow trout   MNGVDRQVLVQTDIQWPNGITLDLIKSRLYWVDSKLHMLCSVDLNGDNRRKVLQSPDYLAHPFALTVFEDRVFWTDGENEAIYGANKFTGSDVITLASNLN 681
Lr8- cutthroat trout MNGVDRQVLVQTDIQWPNGITLDLIKSRLYWVDSKLHMLCSVDLNGDNRRKVLQSPDCLAHPFALTVFEDRVFWTDGENEAIYGANKFTGSDVITLASNLN 681
Lr8- chicken         MNGFDRQQLVTTEIQWPNGIALDLVKSRLYWLDSKLHMLSSVDLNGQDRRLVLKSHMFLPHPLALTIFEDRVFWIDGENEAVYGANKFTGAELVTLVNNLN 706
Lr8+ human           MNGFDRRPLVTADIQWPNGITLDLIKSRLYWLDSKLHMLSSVDLNGQDRRIVLKSLEFLAHPLALTIFEDRVYWIDGENEAVYGANKFTGSELATLVNNLN 685
                     ***.**: ** ::*******:***:*.****:*******.******::*. **:*   * **:***:*****:* ***:**:* ******::: **..***

Lr8+ SFE             EPQDIIVYHELIQLSGTNWCNEKGDNGGCAYMCLPAPQINKHSPKYTCVCPQGQDLATDGQRCKPEPSAAPKDDGKMRTRPPPPSAMPTEPSKNDGKMQTR 785
Lr8- SFE             EPQDIIVYHELIQLSGTNWCNEKGDNGGCAYMCLPAPQINKHSPKYTCVCPQGQDLATDGQRCKPEPSAAPKDDGKMRTRPPPPS---------------- 769
Lr8- white perch     DPQDIIVYHELIQLSGTNWCAEKGVNGGCSYMCLPAPQINKHSPKYTCVCPEGQELAADGLRCRPEANV-------------------------------- 752
Lr8- blue tilapia    DPQDIIVYHELIQLSGTNWCLEKGENGGCSYMCLPAPLINKHSPEYTCVCPEGQELTADGLRCRPEANV-------------------------------- 752
Lr8- rainbow trout   EPQDIIVYHELIQLSGTNWCNEKGLNGGCAYMCLPAPQINKYSPKYTCACPQDQTLASDALHCRPEA----------------------------------- 746
Lr8- cutthroat trout EPQDIIVYHELIQLSGTNWCNEKGLNGGCAYMCLPAPQINKYSPKYTCACPQDQTLASDALHCRPEA----------------------------------- 746
Lr8- chicken         DAQDIIVYHELVQPSGRNWCEENMVNGGCSYLCLPAPQINEHSPKYTCTCPAGYFLQEDGLRCG-------------------------------------- 768
Lr8+ human           DAQDIIVYHELVQPSGKNWCEEDMENGGCEYLCLPAPQINDHSPKYTCSCPSGYNVEENGRDCQ--------------------STATTVTYSETKDTNTT 766
                     : *********:* ** *** *.  **** *:***** **.:**:*** ** .  :  :.  *
                                           <------------------- EGF C ---------------->                     <- O-LINKED SUGAR

Lr8+ SFE             PVLPTASKKPKVVPSPVTAEGNVSTSIHEVNSSAKGSAAAWAILPVLLLAMAAAGGYLMWRNWQLKNKKSMNFDNPVYLKTTEEDLNIDISRHTSTVGHTY 886
Lr8- SFE             -------------------EGNVSTSIHEVNSSAKGSAAAWAILPVLLLAMAAAGGYLMWRNWQLKNKKSMNFDNPVYLKTTEEDLNIDISRHTSTVGHTY 851
Lr8- white perch     -----------------------STS-IQVDSTARGSAAAWAILPVLLLAMAAAGGYLMWRNWQLKNQKSMNFDNPVYLKTTEEDLNIDITRHGANVGHTY 831
Lr8- blue tilapia    -----------------------STS-IQVNSTARGSAAAWAILPVLLLAMAAAGGYLMWRNWQLKNQKSMNFDNPVYLKTTEEDLNIDITRHGANVGHTY 831
Lr8- rainbow trout   ---------------------NSSTSIHEVNFTARGSTAAWAILPVLLLAMAAAGGYLMWRNWQLKNKKSMNFDNPVYLKTTEEDLNIDISRHTSNIGHTY 829
Lr8- cutthroat trout ---------------------NSSTSIHEVNFTARGSTAAWAILPVLLLAMAAAGGYLMWRNWQLKNKKSMNFDNPVYLKTTEEDLNIDISRHTSNIGHTY 829
Lr8- chicken         -------------------GFNISSVVSE--VAARGAAGAWAVLPILLLVTAALAGYFMWRNWQHKNMKSMNFDNPVYLKTTEEDLTIDIGRHSGSVGHTY 850
Lr8+ human           EISATSGLVPG--------GINVTTAVSEVSVPPKGTSAAWAILPLLLLVMAAVGGYLMWRNWQHKNMKSMNFDNPVYLKTTEEDLSIDIGRHSASVGHTY 860
                                           ::   :     :*::.***:**:***. ** .**:****** ** *****************.*** ** ..:****
                     DOMAIN ----------->                  <--- TRANSMEMBRANE --->

Lr8+ SFE             PAISVVNTEDDLS-----   899
Lr8- SFE             PAISVVNTEDDLS-----   864
Lr8- white perch     PAISIVSTDDDLS-----   844
Lr8- blue tilapia    PAISIVSTEDDLS-----   844
Lr8- rainbow trout   PAISVVNTEDDCHNQPSK   847
Lr8- cutthroat trout PAISVVNTEDDLS-----   842
Lr8- chicken         PAISVVSTDDDML-----   863
Lr8+ human           PAISVVSTDDDLA-----   873
                     ****:*.*:**
```
