# Supplementary material for: Lipoprotein receptors in ovary of eel, Anguilla australis: molecular characterisation of putative vitellogenin receptors
Source: Fish Physiol Biochem. 2023 Jan 17;49(1):117–37. doi: 10.1007/s10695-023-01169-6 (PMC9935665; doi:10.1007/s10695-023-01169-6)
Supplement: Supplementary file 1 — Supplementary file1 (ZIP 631 KB) [file 10695_2023_1169_MOESM1_ESM.zip › Online Resource 3.pdf]

Fish Physiology and Biochemistry

“Lipoprotein receptors in ovary of eel, *Anguilla australis*; molecular characterisation of putative vitellogenin receptors”

Lucila Babio*; Erin L. Damsteegt; and P. Mark Lokman.

Department of Zoology, University of Otago, Dunedin, New Zealand.

*Corresponding author (e-mail: lucilababio@gmail.com). Department of Zoology, University of Otago, 340 Great King Street, P.O. Box 56, Dunedin 9054, New Zealand.

Online Resource 3 *Anguilla australis* (shortfinned eel, SFE) Lrp13 aligned with white perch (AHJ60091.1: Reading et al. 2014), cutthroat trout (ALD16281: Mushirobira et al. 2015), greater amberjack (QEL09659.1: Pousis et al. 2019), and yellow croaker (ASS77301.1: Gao et al. 2020) Lrp13 sequences. The signal peptide, the LDLa repeats, the EGF-like repeats, and the transmembrane region are demarcated. The conserved sequences DxSDE and F/YWxD are highlighted. Alignment was performed using Clustal Omega v1.2.4. The “*” represents fully conserved residues, “:” indicates residues with strong similar properties, and “.” represents residues with weak similar properties

```
Lrp13 SFE               MDLLVILCLIALSNSGG--------------------------------------------------------------------------------- 17
Lrp13 cutthroat trout   MAFYLLLSISILEISVLSVAVQTPLKCNLGTKPCKDGSECILYQHVCDGEVDCRDGSDEEDCTVTCTKGQFLCAHGKKCIDQRQVCDGVAQCQDRSDE 98
Lrp13 greater amberjack MGGWLFLFVLLQLSEFL--------------------------------------------------------------------------------- 17
Lrp13 yellow croaker    MGGRLFLCAVLLQLSGF--------------------------------------------------------------------------------- 17
Lrp13 white perch       MGGRLFLCAVLMQLSGP--------------------------------------------------------------------------------- 17
                        *     *
                        <-SIGNAL PEPTIDE>         <--------------- LDLa -------------><---------------- LDLa -------------

Lrp13 SFE               -------------------------------------------------------------------------------------------------- 17
Lrp13 cutthroat trout   VDCLEYMEGCAHHCDKTRCLPDTFLCDGEEDCLDGTDEADCDSDSDGHKNPDEVSENDSSNYKGITTMSAPAPLKCPFGMKPCQDKTECVLYSHVCDG 196
Lrp13 greater amberjack -------------------------------------------------------------QAEPVVSAGSSPLNCGLGSKRCKDGSECVLYSHVCDG 54
Lrp13 yellow croaker    --------------------------------------------------------------------------------------------------
Lrp13 white perch       --------------------------------------------------------------------------------------------------
                        -->      <----------- LDLa ------------>                                    <---------- LDLa -----

Lrp13 SFE               -------------------------------------------------------------------------------------------------- 17
Lrp13 cutthroat trout   DADCKDGSDEETCSLECENGQFQCAHGKKCIDQRQVCDGMAQCQDRSDEMHCLKPMEGCAHHCDNKTRCLPDTFLCDGERDCLDGTDEANCDSDSTDD 294
Lrp13 greater amberjack EPDCRDGSDEEDCASGCNGDQFQCAHRKKCIDKDQVCDGVPQCQDRSDELHCMKQTEGCVHHCDSKSRCLPASFLCDGERDCLDGADEANCEDQEDSG 152
Lrp13 yellow croaker    -------------------------------------------------------------------------------------------------- 17
Lrp13 white perch       -------------------------------------------------------------------------------------------------- 17
                        ----------->    <------------- LDLa -------------->       <------------- LDLa ----------->
```

```
Lrp13 SFE               -----------LSDLDGAEAAEADTPAPQRCTIGFQLCKDGSECVLIRNLCDGEEDCKDGSDEEDCSVGCETEQFQCAHGKKCIDKTEVCDGVAQCQD 104
Lrp13 cutthroat trout   HKNHRYFVAEDYDGNNEGNATSMSAPAPLKCTFGTKPCKNNIECVLYSHVCDGEADCKDGSDEEECSLECGSGQFQCAHGKKCIDQRQVCDRVAQCQD 392
Lrp13 greater amberjack YEKEEK------TSTTPEPAVSDGPSTIIKCPLGAIPCMNKVECILYSHVCDGEADCRDGSDEEECSSACEADQFQCAHGKKCIEQGQVCDGVAQCQD 244
Lrp13 yellow croaker    ------------------SQVVSAGSSPLKCAVGNKPCSDGSECVLYNYVCDGEPDCKDGSDEEDCASECNEDQFQCAHGKKCIEQGQVCDGVSQCQD 97
Lrp13 white perch       ------------------LLVVSGGSSPLKCVLGSKLCQDGSECVLYNHVCDGEADCKDGSDEEECSLACEKDQFQCAHGKKCIEQSQVCDGVSQCQD 97
                                            .     :  :* .*   * :  **:*   :**** **:******:*:  *   ***********:: :*** *:****
                                                      <-------------- LDLa 1 ------------>   <------------ LDLa 2 --------

Lrp13 SFE               RSDETDCWKPTPSCALRCDNKTRCVPESFLCDGERDCLDGSDEASCADEACGSGDFRCGSGQCLSEHVRCDGHADCPDRSDEKGCGRASDCPTQRRCP 202
Lrp13 cutthroat trout   RSDEMHCLKPMEGCAHHCDNKTRCLPDTFLCDGERDCLDGTDEANCADESCGSGEFRCTSGQCVSISMRCDGHPDCWDHSDEESCHKPPQCTTNRRCP 490
Lrp13 greater amberjack RSDELGCAKHMEDCAHQCDDKSRCIPNSFLCDGEMDCWDGSDEANCADEDCSATEFKCTSGQCVSATMRCDGHPDCWDHSDEESCTKPPVCTTKHRCL 342
Lrp13 yellow croaker    RSDEMGCAKQMEGCAHQCDNKSRCIPNSFLCDGERDCGDGSDEKNCVDEGCSATDFKCTSGQCVLATMRCDGHPDCWDRSDEESCTNAPACTTKHRCP 195
Lrp13 white perch       RSDELGCAKHMEGCAHQCDDKTRCIPNSFLCDGEKDCWDSSDEANCEDQGCSATEFKCTSGQCVSATMRCDGHPDCWDRSDEESCTNAPACTTKHRCT 195
                        ****  * *   .** :**:*:**:*::****** ** *.:** .* *: *.: :*:* ****:   :***** ** *:***:.* .   * *::**
                        ------>      <----------- LDLa 3 ----------->     <------------ LDLa 4 ------------>            <-

Lrp13 SFE               RSGECLLDEWICDGEEDCRDGTDEKDCKVAQIKCGEFQWSCASKIECVPTSWRCDGVKDCKDDSDEIGCPGV-KCPSHQFQCGT-GECLDPSLVCDGT 298
Lrp13 cutthroat trout   QSQECLLEEWVCDGEQDCKDGSDEENCEMVQLKCGAFQWACTSKSQCVPKAWRCDGTKDCADDSDEAGCGQVATCPSHQFQCASTSECLDMALVCNSV 588
Lrp13 greater amberjack QSKECLVQEWICDGDQDCKDGTDEKDCPVAPLTCGEFQWSCKSKTKCIPSAWRCDGVKDCNDGSDETECGVV-ACLPHQFQCGS-QECLDPVLVCNNI 438
Lrp13 yellow croaker    KSKECLVQEWICDGDQDCKDGTDEKDCPLAPLNCAEFQWSCKSKTECIPTAWRCDGMKDCDDGSDETECGVV-TCLPHQFQCGS-QECLDPSLVCNGI 291
Lrp13 white perch       QSKECLLQEWICDGDQDCKDGTDEKDCPMVPLNCGEFQWSCKSKTKCIPTAWRCDGMKDCDDGSDETECTMV-TCLPHQFQCGS-QECLDPVLVCNGI 291
                        :* ***::**:***::**:**:**::* :. :.*. ***:* ** :*:*.:***** *** *.***  *  *  *****.:  ****  ***:.
                        -------- LDLa 5 --------->       <------------- LDLa 6 ------------>      <---------- LDLa 7 -----

Lrp13 SFE               SNCPDGSDEGGACASSNCSGPKAPRCTQNCYRMPQGMRCACMAGFRLQTDAVSCVDVNECEETLPRACSHTCLNTQGSYECSCHPGYLLEPDARSCKI 396
Lrp13 cutthroat trout   RNCADGSDEGGDCK-TTCP--DKAICVQNCYSTPQGTRCGCKAGCQLDEDAVSCGDIDECVGGRQGVCSHSCVNTLGSFHCHCNPGYLLESDGRHCKI 683
Lrp13 greater amberjack TNCADGSDEGGSCQ-INCAEADNSRCSQSCYSTPQGTRCHCTAGFSLMEDGLTCADIDECDGQSPAVCSQLCINTPGSYRCDCHPGYIMEAGGHNCKI 535
Lrp13 yellow croaker    TNCADGSDEGGSCR-INCAEADSRRCSQNCYNTPQGTRCRCAAGFKLMEDGLTCADIDECE--SPGVCSQLCYNTPGSYQCDCHPGYIIEADGRQCKI 386
Lrp13 white perch       TDCADGSDEGGSCQ-INCAEADRSRCTQGCYNTPQGVRCRCAAGFRLMEDRLTCADIDECEGWSPGVCSQLCINTPGSYQCGCHPGYIMEASGHQCKI 388
                         :* ********    .*   .   * *.**  *** ** * **  *  * ::* *::**      .**: * ** **:.* *:***::* ..: ***
                        ----------->     < -------------- EGF A ------------->     <------------- EGF B -------------->

Lrp13 SFE               TAGEPVLLAAVQHELLLFSLRNATLDVLFASGKGAVLSLDYDWRERTVFWVSRDADSIKWISLDQRHMGTMIKGVKLDCIAVDWVGRNLYWTDGVGG- 493
Lrp13 cutthroat trout   M-GEPYLLASVQTDLFLVGLRSSSLDVLVSSAKKAILSVDYDWRDQRVFWVSLDSESIKWSSLDQKKRGTLFKGIKSDCIAVDWVGRNLYWIDGIGGG 779
Lrp13 greater amberjack T-GEPLLLSSVQTDLFLVGLRSGSVDVLSSSAKKAILSVDYDWREQRVYWVSLDSENIRWSSLDQKTTGTLIKGIRADSVAVDWLGRNLYWIDGVNS- 630
Lrp13 yellow croaker    T-GEPFLLASIQTDLFLLGLRSGSLDVLSSSAKKAILSLDYDWREQRVFWVSLDTESIRWSSLDQKTTGTLIKGVRADSVAVDWLGRNLYWIDGVNS- 481
Lrp13 white perch       T-GEPFLLASIQTDLFLFGLRSGSLDVLSSTARKAILSLDYDWKEQRVFWVSLDTEGIRWSSLDQKTTGTLIKGVRADSVAVDWLGRNMYWIDGVNS- 483
                          *** **:::* :*:*..**..::*** ::.: *:**:****::: *:*** *::.*:* ****:  **::**:: *.:****:***:** **:..
```

```
Lrp13 SFE                  QILATGLNTTATDSWSFTVVLDEDLEQPHSLVLLPQKGLMFWSEIGSEPQIERAGMDGSGREVVVSRSLSWPASLSVDPLADRVYWTDEKLKCIGSAT 591
Lrp13 cutthroat trout      RIIAIGLNSTITSALDLTVILDKEFKQLLSLALLPQKGLLFWSEISNEAKIERAGMDGSERRVVVSHSLSWPGSLAVDPIGERLYWTDKKLGCIGSAT 877
Lrp13 greater amberjack   QIVAIRLATGTLESLDHSVILDEDLDQPRSLALLPQKGLMFWTEIGNVVKIERAGMDGSERRAVVNSSLGWPGGVAVDTISDRVYWTDERLGAIGSAT 728
Lrp13 yellow croaker      KIVAIRLATTTVNSLNHSVILDEDLDQPRSLALLPQKGLMFWTEIGNVVKIERAGMDGSERTAVVNSSLGWPGGVAVDTISDRVYWTDERLRAIGSAT 579
Lrp13 white perch         QIVAIRLATTTVKSLDHSVILDEDLDQPRSLALLPQKGLMFWTEIGNVVKIERAGVDGSERRAVVNSSLGWPGSVAVDTIPDRVYWTDERLRAIGSAA 581
                           :*:*  * :   .: . :*:**:::.*  **.*******:**:**..  :*****:*** * .**. **.**..::** : :*:****::* .****:

Lrp13 SFE                  FDGGDIKIIQLMEMSSPFSVMVFNDRIYWSDTKRRTIQSAHKDSGKDREVLLKRLGQPFGLKVIHELLQPNGGEPCAERACSHLCLLAPGPRGVCHCP 689
Lrp13 cutthroat trout      LDGGDVKILQLTETTNPFSVTVFNDMLYWSDTKRGTIQGANKITGKNCKVLLKRPAQPFGLKVIHPLLQTSTDSPCEKLHCSHLCVLAPGPKGVCKCP 975
Lrp13 greater amberjack   LDGDDIQILQMKETTNPFSLAVFNNMLYWTDAKKRVVQAAHKISGKNRQVLLKRPRQPFAVKIIHSLLQMGNQSPCEKMDCSHMCVLAPGPKAVCKCP 826
Lrp13 yellow croaker      LDGDDIRILQMKETTNPFSLAVFNDILYWSDAKKRVVLAAHKISGKNRQVLLKRPRQPFGVKIIHPLLQMGIEGLCEKTHCSHMCVLAPGPKAVCKCP 677
Lrp13 white perch         LDGDDIQILQMKGTTNPFSVAVFNDVLCWSDTKKRVVLAAHKISGKNRKVLLKRQRQPFGVKIIHPLLQMGIESPCEKMDCSHMCVLAPGPKAVCKCP 679
                           :**.*::*:*:   :.***: ***: : *:*:*: .: .*:* :**: :*****  ***.:*:** ***  .     * :  ***:*:*****:.**:**
                                                                                                            <-------- EGF C -------

Lrp13 SFE                  SGLLLAADGTTCAAPAPEDDSFLLLLSPTAVTQIYLRGVRGGVRLQGWPEHRAVSLGRVNEPTALDLAVRDRRLYLADAGQGAIGLYGLGAAPALQGV 787
Lrp13 cutthroat trout      SGLLLAEDGLNCSNL--VNSAFLLVLSPTVVTQIYLQTMLSAVGLKTWPEHLSLPLANVNEADILDYTLRDKMLYLADSGQSSVGLFKLK------ET 1065
Lrp13 greater amberjack   SGLSLAEDGLTCSSL--VNSAFLLILSPSTVTQIYLQSRHTAAELKGWPEHLALQVPSVNEAAIMDYNLRYHTLFLTDDGTTSLSSFKLK------DS 916
Lrp13 yellow croaker      SGLLLAENGLTCSSL--VNSAFLLMLSPSTVTQIYLQSRHTTAELNGWPEHLALQVPSVNEAAIMDYSLHDHTLFLTDDGTTSLSSFKLK------DS 767
Lrp13 white perch         SGLLLAKDGLTCSSL--VNSAFVLMLSPSTVTQIYLQSRHTAAELKGWPEHLALQVHSVNEAAIMDYSLRDRTLFLTDDGTTSLSSFKLK------DA 769
                           *** ** :* .*:     :.:*:*:***:.******:     . *: **** :: :  ***   :*  :: : *:*:* *  ::. : *
                           ----------->

Lrp13 SFE                  ALEPQGVAVQLEGETVAALALDWVTLNVYWSGSERPRIRVTAPGGRRSATLLHKGVQSPASLALHPPSGWLCFADLGRPDHPDPARLECAFMDGRNRT 885
Lrp13 cutthroat trout      SLVPRGQFLQLKGDTVTALALDWITLNVYWSSTKQSRLQVTSSNGEHTAVLIDM--GSLKSIALHPLSGRLCFAKQ-----GEGAHVECAHMDGGKRA 1156
Lrp13 greater amberjack   DLTSQGQLLKLLGDAISAMALDWVTLSVYWSSNKQRRLQVTSITGAYTAVLIKEGIGRVESIALHPPSGRVCFTNLARQDIGTVATVECANMDGAERR 1014
Lrp13 yellow croaker      DLSPQGRLLKLLGDTITAMALDWVTFNVYWSSNKQPRLQVTSITAAHTAVLIKEGISRVKSIALHPPRGRVCFTNLGLQGTGTVATVECANMDGAGRS 865
Lrp13 white perch         DLSSQGQLLKLLGDTITAMALDWVTLNIYWSSNKQPRLQVTSITAAHAAVLIKEGISRAESIALHPPSGRVCFTNLVLQGKGTVATVECANMDGAGRN 867
                            *  :*  ::* *::::*:****:*:.:***..:: *::**:  .  :*.*:.       *:****  * :**:.         * :*** ***  *

Lrp13 SFE                  LLWKRAATPTSLSFSDDGAQLYWADIDTGVIASIRLDGSGYRERQTGGGSIQAFAYGDGMLFWATRNDTSKVWFGDGLKSKTLWFEVKTEVVSLKAYG 983
Lrp13 cutthroat trout      QVWKDAVQPTSLTFSNDGGEIYWADIGAGVIGSVRVDGSGYIEFTTGDG-LIAFALSNSMLLWVTDRDTTEVWYRDDQLIKTLWFEVNTEVVSLKAYS 1253
Lrp13 greater amberjack   VMWKDAVQPTSLVFSSNGDTIYWADSGLGTIGYVQLDGSGYRELKAGDG-LAAVALSDDTLLWMTVNDKTRLWYRDEQQQNKLWFEVGTEVVSLKAFS 1111
Lrp13 yellow croaker      VVWKDAVQPTSLAFSTNGDTIYWVDTGLGTVSSVQLDGSGYRELKAGDG-LAAVALSDDALLWMTVSDKTRIWYRDEQQQNKLWFEVGTEVVSLKAFS 962
Lrp13 white perch         VVWKDAVQPTSLVFSSNGDTIYWADTGLGTIGSVQLDGSGYRELKTGDG-LVAVALSDDTLLWMTVSDKTRLWYRDEQQASKLWFEVGTEMVSLKAFS 964
                            :** *. **** ** :*  :**.* . *.:. ::****** *  :*.* : *.* .:. *:* *  *.:.:*: *    ..***** **:*****:.

Lrp13 SFE                  KGSQKGSNGCSHKNGGCSHLCLAFPGGRTCRCAQDHRPVNTTDCAPD--CPPDSRPCRDGRSCTPLSKVCDGHPDCADQSDEDCESGLNSSV---KTP 1076
Lrp13 cutthroat trout      KSSQMGFNFCSDGNGDCSHFCLAVPGGRTCRCAHGHRPVNATHCALDQHCPAGSRPCLDGHTCLPLEKFCDGHPDCLDTSDENCVHLKGQSEVQSKAP 1351
Lrp13 greater amberjack   KSSQTGSNQCTENNGNCQHLCLATPGGRTCKCGHDHVLVNATHCSPEQGCPAGTRPCLDQVSCQPVEKFCNGRVDCSDHSDENCVNLKQWSGIKVLAP 1209
Lrp13 yellow croaker      KSSQTGSNQCTENNGNCQHLCLATPGGRTCKCAHDHILVNATHCSPEQHCPDGSRPCLDQLSCQPVEKFCNGHTDCYDHSDENCVTLKQWSGAKVHAP 1060
Lrp13 white perch         NSSQTGSNQCTD-NGNCQHLCLATPGGRTCKCAHDHILVHDTHCNPEQRCPSGTRPCLDQLSCQPIEKFCNRHVDCYDHSDENCVSVKQWSEAKVPAL 1061
                           :.** * * *:. **.*.*:*** ******:*.:.*  *: *.*  :  ** .:*** *  :* *:.*.*: : ** * ***:*       *     :
                                 <------------- EGF D ------------>     <------------ LDLa +1 ----------->
```

```
Lrp13 SFE               ADPQDGTLPPVSVSRATPVLATPVPAMPTPPPEAGRVPVESLD-AEPCGERLCNGRGGCVSQNGEAVCECEAGFSGEFCQDGAFAALRTPLAYAAIAL 1173
Lrp13 cutthroat trout   TLLPSPSIPT------SADL------GSTTFLDNSG-LVRNLDVKQQCSEKRCNGNGECVETNGGTSCPCGLGYSGDSCQDQLANPMQGPLIYGAIGF 1436
Lrp13 greater amberjack TQPRSSSPPPPPLPPLSETN------DLNTTLNVSS-LLMNLD-AQQCSQRHCSGNGRCVDNSGDTVCVCSLGYSGDSCTDHLLQTVQRPLVYGAAVL 1299
Lrp13 yellow croaker    TRPLSSSTPPSSFPALSEVA------SPNNTLNVGG-QLMNLD-TQECSQKLCSGNGRCVVINGDTACVCSLGYSGDSCQDHLLKTLQNPFVYGAAGL 1150
Lrp13 white perch       TWAHNSSPPTFPLPGLSGVS------GLNTTLNESS-QLRNLA-TQDCSQKHCSGNGRCVKNNGNSACVCSLGYSGDSCQDHLLKTMQGPIVYSAAGL 1151
                        :   . : *       :           .   : .   : .*   : *.:: *.*.* **  .* : * *  *:**: * *     :: *: *.*  :
                                                       <----------- EGF E ------------->         <- TRANS-

Lrp13 SFE               VAAVIVVGVVLGVRKRRMTQ---RRERTAAKETSLMDMEKRTEESSNQNG--KKDTCDPTEELPPL-  1234
Lrp13 cutthroat trout   CAAIVVISVLVAVVR-RKTANARRASPV-VKQTSMTDWGKHGDSP-FTQHS-PDDINFSEEVASSVG  1500
Lrp13 greater amberjack GAGVLVISVLAVVIKRKKNANMRRASPAAMKDTSMTDLEKKAETAPPTTA--PVDTEKPEEAVSSVD  1365
Lrp13 yellow croaker    CAGMVVIAVMAVVVKRRKSANTRRASPAAVKETSMTDLENKAETTQSTQISPAPNTDDPEEVVSSVD  1218
Lrp13 white perch       CAGVVVIAVMAVVVKRRKSANGRRARPAAVKETSMTDLENKAETTPSKQPSPV-DTDKPEEVASSVD  1218
                         *.::*:.*:  * : : .    *   .  *:**: *  :: :           :     *    :
                        MEMBRANE ---->
```
