# Supplementary material for: Lipoprotein receptors in ovary of eel, Anguilla australis: molecular characterisation of putative vitellogenin receptors
Source: Fish Physiol Biochem. 2023 Jan 17;49(1):117–37. doi: 10.1007/s10695-023-01169-6 (PMC9935665; doi:10.1007/s10695-023-01169-6)
Supplement: Supplementary file 1 — Supplementary file1 (ZIP 631 KB) [file 10695_2023_1169_MOESM1_ESM.zip › Online Resource 4.pdf]

*Fish Physiology and Biochemistry*

“Lipoprotein receptors in ovary of eel, *Anguilla australis*; molecular characterisation of putative vitellogenin receptors”

Lucila Babio*; Erin L. Damsteegt; and P. Mark Lokman.

Department of Zoology, University of Otago, Dunedin, New Zealand.

*Corresponding author (e-mail: lucilababio@gmail.com). Department of Zoology, University of Otago, 340 Great King Street, P.O. Box 56, Dunedin 9054, New Zealand.

**Online Resource 4** Fragment of *lr8+* and *lr8-* variants from the shortfinned eel, *Anguilla Australis,* aligned with corresponding forward (FW) and reverse (RV) qPCR primers. To detect the *lr8+* variant, the qPCR primers were designed to target the putative *O*-linked sugar domain, whereas to detect the *lr8-* variant, the RV primer was designed to span the exon boundary (indicated with arrows) corresponding to the position of the absent putative *O*-linked sugar domain. The putative *O*-linked sugar domain is highlighted in yellow and the red bold text corresponds to the qPCR primers from the *lr8-* variant

```
lr8+             TCCAGCTGTCAGGGACAAACTGGTGTAACGAGAAAGGAGATAATGGCGGCTGTGCCTACATGTGCCTGCCGGCCCCCCAGATCAACAAGCACTC 2258
lr8-             TCCAGCTGTCAGGGACAAACTGGTGTAACGAGAAAGGAGATAATGGCGGCTGTGCCTACATGTGCCTGCCGGCCCCCCAGATCAACAAGCACTC 2258
lr8+ FW primer   ---------------------------------------------------------------------------------------------- 0
lr8+ RV primer   ---------------------------------------------------------------------------------------------- 0
lr8- FW primer   -----------------------------------GGAGATAATGGCGGCTGTG---------------------------------------- 19
lr8- RV primer   ---------------------------------------------------------------------------------------------- 0

lr8+             CCCCAAGTACACCTGCGTCTGCCCCCAGGGGCAGGACCTGGCCACCGATGGTCAGCGCTGCAAACCCGAGCCTTCAGCAGCCCCAAAGGATGAT 2352
lr8-             CCCCAAGTACACCTGCGTCTGCCCCCAGGGGCAGGACCTGGCCACCGATGGTCAGCGCTGCAAACCCGAGCCTTCAGCAGCCCCAAAGGATGAT 2352
lr8+ FW primer   ---------------------------------------------------------------------------------------------- 0
lr8+ RV primer   ---------------------------------------------------------------------------------------------- 0
lr8- FW primer   ---------------------------------------------------------------------------------------------- 19
lr8- RV primer   ---------------------------------------------------------------------------------------------- 0

lr8+             GGGAAAATGAGAACACGCCCCCCTCCTCCTTCAGCCATGCCTACGGAGCCCTAAAGAATGATGGGAAGATGCAGACACGGCCCGTTCTGCCTAC 2447
lr8-             GGGAAAATGAGAACACGCCCCCCTCCTCCTTCAG------------------------------------------------------------ 2386
lr8+ FW primer   -----------------------------------------TACGGAGCCCTCAAAGAATG--------------------------------- 20
lr8+ RV primer   ---------------------------------------------------------------------------------------------- 0
lr8- FW primer   ---------------------------------------------------------------------------------------------- 19
lr8- RV primer   ------------------------CCTCCTTCAG------------------------------------------------------------ 9
                                                   ←|→
```

```
lr8+             AGCCATCAAAGAAACCAAAAGTGGTCCCCAGTCCAGTCACTGCTGAGGGGAACGTCAGCACCTCGATCCATGAGGTG  2523
lr8-             ---------------------------------------------AGGGGAACGTCAGCACCTCGATCCATGAGGTG  2418
lr8+ FW primer   -----------------------------------------------------------------------------  20
lr8+ RV primer   -----------------------------AGTCCAGTCACTGCTGAGGG----------------------------  20
lr8- FW primer   -----------------------------------------------------------------------------  19
lr8- RV primer   ---------------------------------------------AGGGGAACGT----------------------  20
                                                            ←|→
                                                       EXON BOUNDARY
```
