# Supplementary material for: Lipoprotein receptors in ovary of eel, Anguilla australis: molecular characterisation of putative vitellogenin receptors
Source: Fish Physiol Biochem. 2023 Jan 17;49(1):117–37. doi: 10.1007/s10695-023-01169-6 (PMC9935665; doi:10.1007/s10695-023-01169-6)
Supplement: Supplementary file 1 — Supplementary file1 (ZIP 631 KB) [file 10695_2023_1169_MOESM1_ESM.zip › Online Resource 5.pdf]

Fish Physiology and Biochemistry

“Lipoprotein receptors in ovary of eel, *Anguilla australis*; molecular characterisation of putative vitellogenin receptors”

Lucila Babio*; Erin L. Damsteegt; and P. Mark Lokman.

Department of Zoology, University of Otago, Dunedin, New Zealand.

*Corresponding author (e-mail: lucilababio@gmail.com). Department of Zoology, University of Otago, 340 Great King Street, P.O. Box 56, Dunedin 9054, New Zealand.

Online Resource 5 a) Relative *actb* transcript abundance, normalised over total RNA, and **b)** relative *eef1a* transcript abundance, normalised over total RNA, in ovary of shortfinned eel, *Anguilla australis*, during artificial maturation. The expression of both genes followed a significant decreasing trend as oocyte development advanced, preventing their use as reference genes (*actb* one-way ANOVA: F = 33.61, df = 5, $p<0.0001$; t-test: t = 1.48; df = 8, $p = 0.2$; *eef1a* Kruskal Wallis: H = 23.1, df = 5, $p<0.001$; Mann-Whitney U test: U = 3.5, $p = 0.06$). Different letters indicate significant differences between groups ($p<0.05$). **c)** Relative *lr8+*, **d)** *lr8-* and **e)** *lrp13* transcript abundance normalised over *actb*. The three putative Vtgrs had stable expressions as no significant differences were found between treatments, neither between controls Week 0 and C. Data are shown as mean ± SEM per Week of treatment and control – C (all groups at n = 5, except for Weeks 8 and 10 at n = 4)

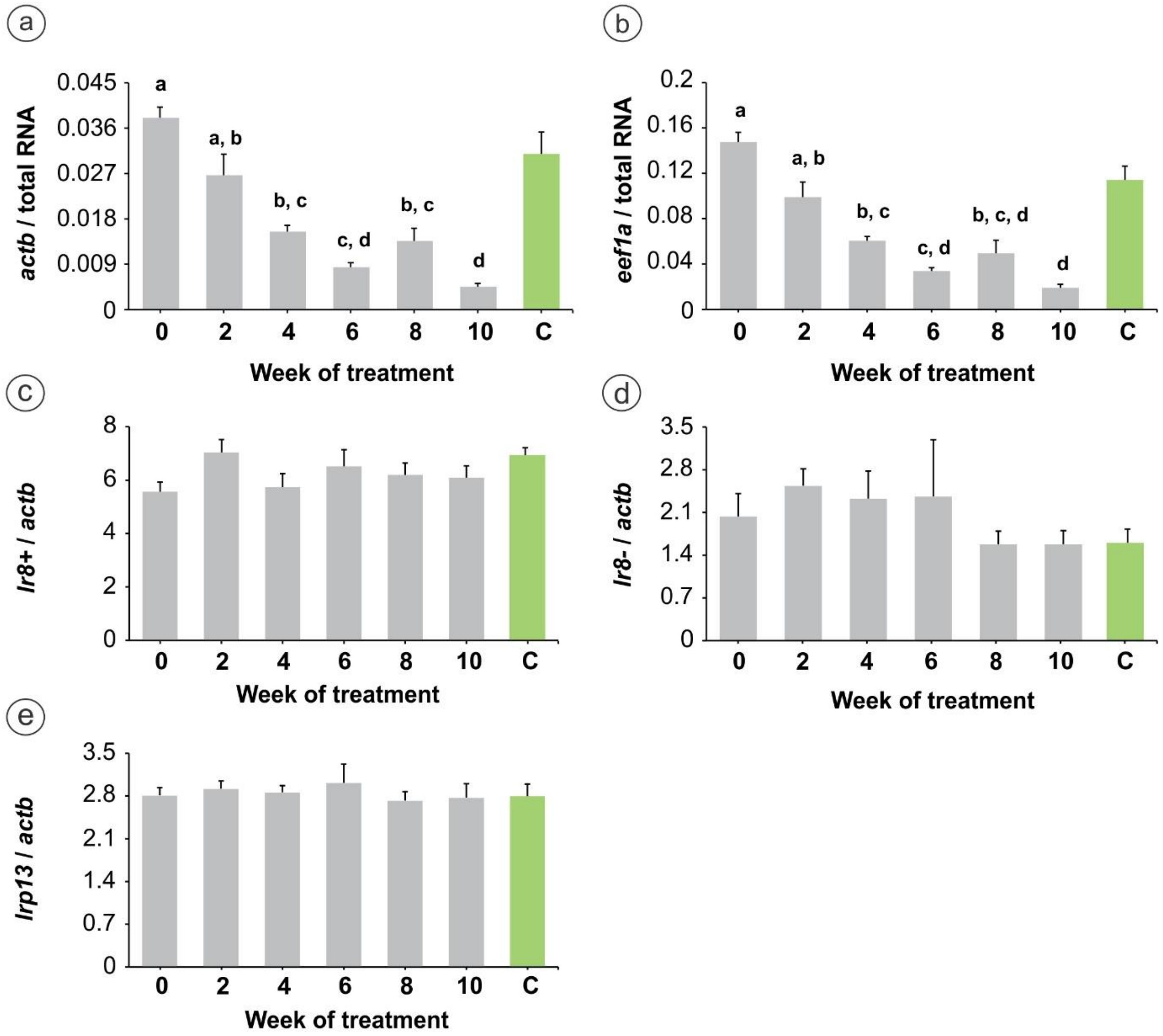
a
actb / total RNA
0.045
0.036
0.027
0.018
0.009
0
a
a, b
b, c
c, d
b, c
d
0
2
4
6
8
10
C
Week of treatment
b
eef1a / total RNA
0.2
0.16
0.12
0.08
0.04
0
a
a, b
b, c
c, d
b, c, d
d
0
2
4
6
8
10
C
Week of treatment
c
Ir8+ / actb
8
6
4
2
0
0
2
4
6
8
10
C
Week of treatment
d
Ir8- / actb
3.5
2.8
2.1
1.4
0.7
0
0
2
4
6
8
10
C
Week of treatment
e
Irp13 / actb
3.5
2.8
2.1
1.4
0.7
0
0
2
4
6
8
10
C
Week of treatment
